# “Intrasellar tumor-to-tumor metastasis: A single center experience with a systematic review”

**DOI:** 10.1007/s11102-024-01441-9

**Published:** 2024-08-14

**Authors:** Guilherme Mansur, Mohammad Bilal Alsavaf, Ludovica Pasquini, Moataz D. Abouammo, Chandrima Biswas, Pavnesh Kumar, Raju R. Raval, Peter Kobalka, Ricardo L. Carrau, Daniel M. Prevedello

**Affiliations:** 1 Department of Neurological Surgery, Wexner Medical Center at The Ohio State University, Doan Hall N 1049, 460 W 10th Ave., Columbus, OH 43210 USA; 2 Department of Otolaryngology-Head and Neck Surgery, Wexner Medical Center at The Ohio State University, Columbus, OH USA; 3 Department of Otorhinolaryngology-Head and Neck Surgery, Tanta University, Tanta, Egypt; 4 Department of Radiation Oncology, Wexner Medical Center at The Ohio State University, Columbus, OH USA; 5 Department of Pathology, Wexner Medical Center at The Ohio State University, Columbus, OH USA

**Keywords:** Tumor-to-tumor metastasis, Pituitary adenomas, Renal cell carcinoma, Prostate adenocarcinoma, PitNET, Collision tumors

## Abstract

**Purpose:**

This study investigates the rare occurrence of tumor-to-tumor metastasis in Pituitary Neuroendocrine Tumors (PitNETs), also known as pituitary adenomas, aiming to enhance understanding of its diagnostic and therapeutic challenges. We report two cases from our institution of tumor-to-tumor metastasis involving PitNETs, followed by a systematic literature review.

**Methods:**

We conducted a comprehensive literature review using PubMed and Google Scholar databases. This review provides insights into patient demographics, clinical presentations, primary tumor origin, management approaches and outcomes.

**Results:**

We identified 38 documented cases of tumor-to-tumor metastasis involving the pituitary gland in the literature. This revealed a diverse range of primary tumor origins, with lung, breast, and renal carcinomas being the most prevalent. Clinical presentations varied, with visual disturbances emerging as the most frequently reported symptom. Surgical interventions predominantly resulted in subtotal resection. Kaplan–Meier survival analysis demonstrated that endoscopic endonasal approaches (EEA) are associated with longer median survival times compared to other surgical methods.

**Conclusion:**

Tumor-to-tumor metastasis to PitNETs must be considered in differential diagnoses of sellar masses. Prompt and accurate diagnosis, coupled with a multidisciplinary treatment strategy, is essential. Our study contributes to the scarce literature on such metastases, providing a foundation for further understanding of this complex pathological entity.

**Supplementary Information:**

The online version contains supplementary material available at 10.1007/s11102-024-01441-9.

## Introduction

The occurrence of multiple, histologically distinct tumors at a single anatomical site is an exceptionally rare event in medical practice, presenting unique challenges in diagnosis, treatment, and prognosis. This is particularly uncommon within the sellar region.

Two mechanisms have been described through which distinct tumors might coexist within the same anatomical region. [1] The first, known as a collision tumor, occurs when two neighboring neoplasms grow towards one another until they eventually converge. The sellar region has seen instances of collision tumors, particularly involving Pituitary Neuroendocrine Tumors (PitNETs), also known as pituitary adenomas, coexisting with craniopharyngiomas [2–15]. Likewise, collision tumors linking PitNETs to meningiomas, gliomas, chondromas, and even inverted papillomas have been reported. [16–34]

The second mechanism is tumor-to-tumor metastasis. This process involves the hematogenous spread of malignant cells from its primary site to a distant distinct primary tumor, such as a PitNET. Throughout our review, we found reports on breast adenocarcinoma[35–41], lung carcinoma [42–52], renal cell carcinoma[53–56], colorectal adenocarcinoma[57–60], gastric carcinoma[49, 61], mediastinal cancer [62], melanoma[63, 64], esophageal cancer [65], pancreas and prostate cancer[66] metastasizing to PitNETs. Metastases from an unidentified primary site have also been reported [38, 51, 61, 67, 68].

This paper explores the phenomenon of tumor-to-tumor metastasis within the sellar region, presenting two cases involving PitNETs as the secondary site of metastasis. Additionally, we provide an extensive review of the existing literature on tumor-to-tumor metastasis to PitNETs, seeking to enhance our understanding of its clinical implications, diagnostic challenges, and treatment considerations.

## Case reports

### Case report #1

A 56 years-old male was referred to our neurosurgery department due to a progressive visual decline over the past two years, which had significantly deteriorated to bilateral blindness. His medical history was notable for renal cell carcinoma, diagnosed 2 years prior, for which he underwent laparoscopic nephrectomy to remove a large right renal mass. His examination revealed pale, dry skin, substantial recent weight loss, and a history of daily smoking.

The patient’s magnetic resonance imaging (MRI) revealed a large, heterogeneous sellar/suprasellar mass, showing diffuse contrast enhancement and signs of intralesional hemorrhages. The lesion was pressing the optic nerves/chiasm and invading the cavernous sinuses and clivus.

Endocrinological evaluation showed hypocortisolism (2.62* μg/dl,* NR 5-25* μg/dl*), hyponatremia (133* mEq/L,* NR 135–145* mEq/L*), hypothyroidism (T4F: 0.64* ng/dL,* NR 0.9–2.3* ng/dL*), hypogonadism (Test < 7* nmol/L*, NR 10–35* nmol/L*), hyperphosphatemia (4.7* mg/dl, NR* 2.5–4.5* mg/dL*) and hypomagnesemia (1.5* mg/dL,* NR 1.7–2.2* mg/dL*). Patient was promptly started on levothyroxine and hydrocortisone.

**Fig. 1:**
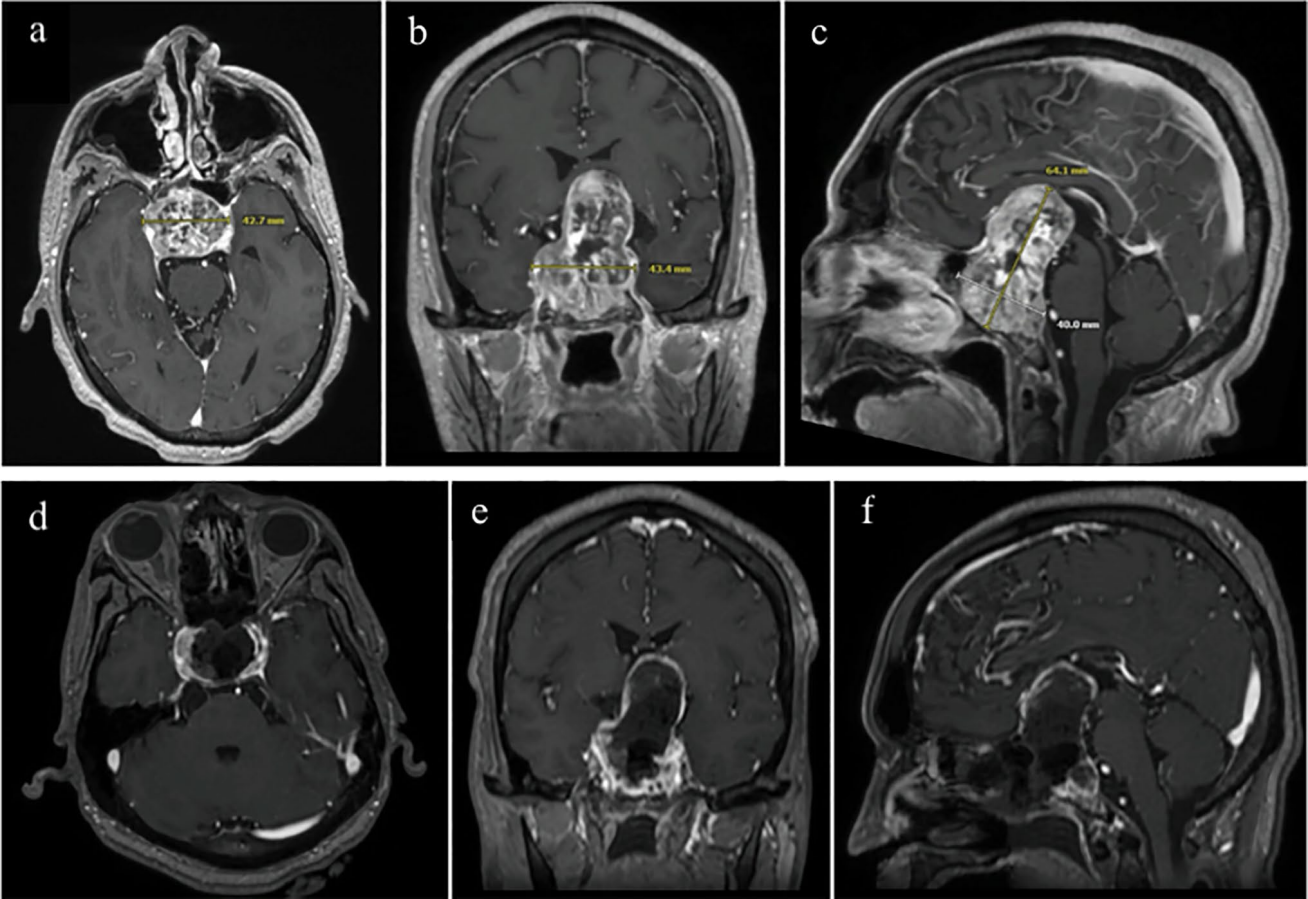
The images show radiological studies of the patient from the first case report. **a**, **b** & **c** Axial, Coronal, and Sagittal cuts from brain MRI (T1 C +) showing large sellar/suprasellar heterogenous mass, measuring 43.3 × 64.1×40mm, with diffuse contrast enhancement and signs of intralesional hemorrhages. It causes significant mass effects over the optic nerves/chiasm as well as invasion over the cavernous sinuses and clivus. **d**, **e** & **f** Axial, Coronal, and Sagittal cuts from intraoperative brain MRI (T1 C +) showing subtotal resection of large sellar/suprasellar mass, with little residual tumor on the right cavernous sinus

He subsequently underwent endoscopic endonasal transsphenoidal/transtuberculum approach for tumor resection (Video 1). Intraoperative MRI post-resection confirmed the absence of apoplexy and showed a subtotal resection with a small residual mass in the right cavernous sinus (Fig. 1).

**Fig. 2:**
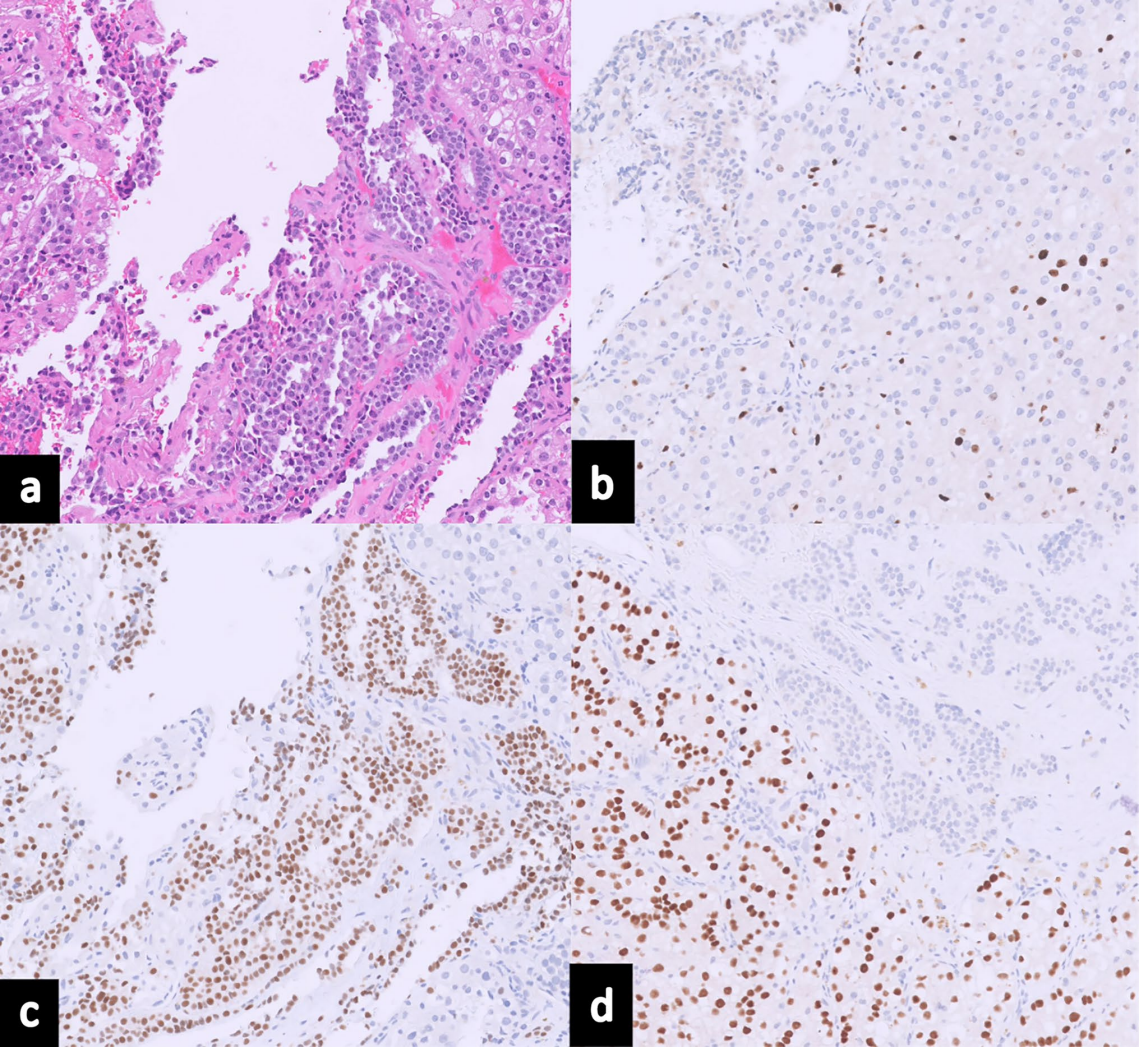
Histopathological Analysis from Surgical Resection in Case Report 1: **a** Hematoxylin and Eosin (H&E) staining demonstrates a dual histological presentation: clear cell renal cell carcinoma is seen in the lower left quadrant with characteristic features such as abundant clear cytoplasm, while the upper right quadrant exhibits a PitNET pattern with epithelioid cells forming expansive lobules. **b** Ki-67 immunostaining, a marker of proliferation, is shown with a low labelling index, indicating 1% of tumor cells. **c** Immunohistochemical staining for Steroidogenic Factor 1 (SF-1) reveals positive staining within the PitNET, confirming its nature. **d** PAX-8 immunostaining, specific for renal lineage, highlights positivity within the metastatic renal cell carcinoma cells

The postoperative period was uneventful, with no improvement of visual deficit, and the patient was discharged on the third postoperative day. The final histopathological examination confirmed the coexistence of renal cell carcinoma metastasis and gonadotroph PitNET (Fig. 2). Details on the pathology can be found in **Supplementary Material 1**. On the last follow-up, 6 weeks after surgery, the patient maintained visual deficits, with no additional complaints. He was receiving radiation for the residual tumor and keeping regular follow-ups with Oncology and Endocrinology.

**Fig. 3:**
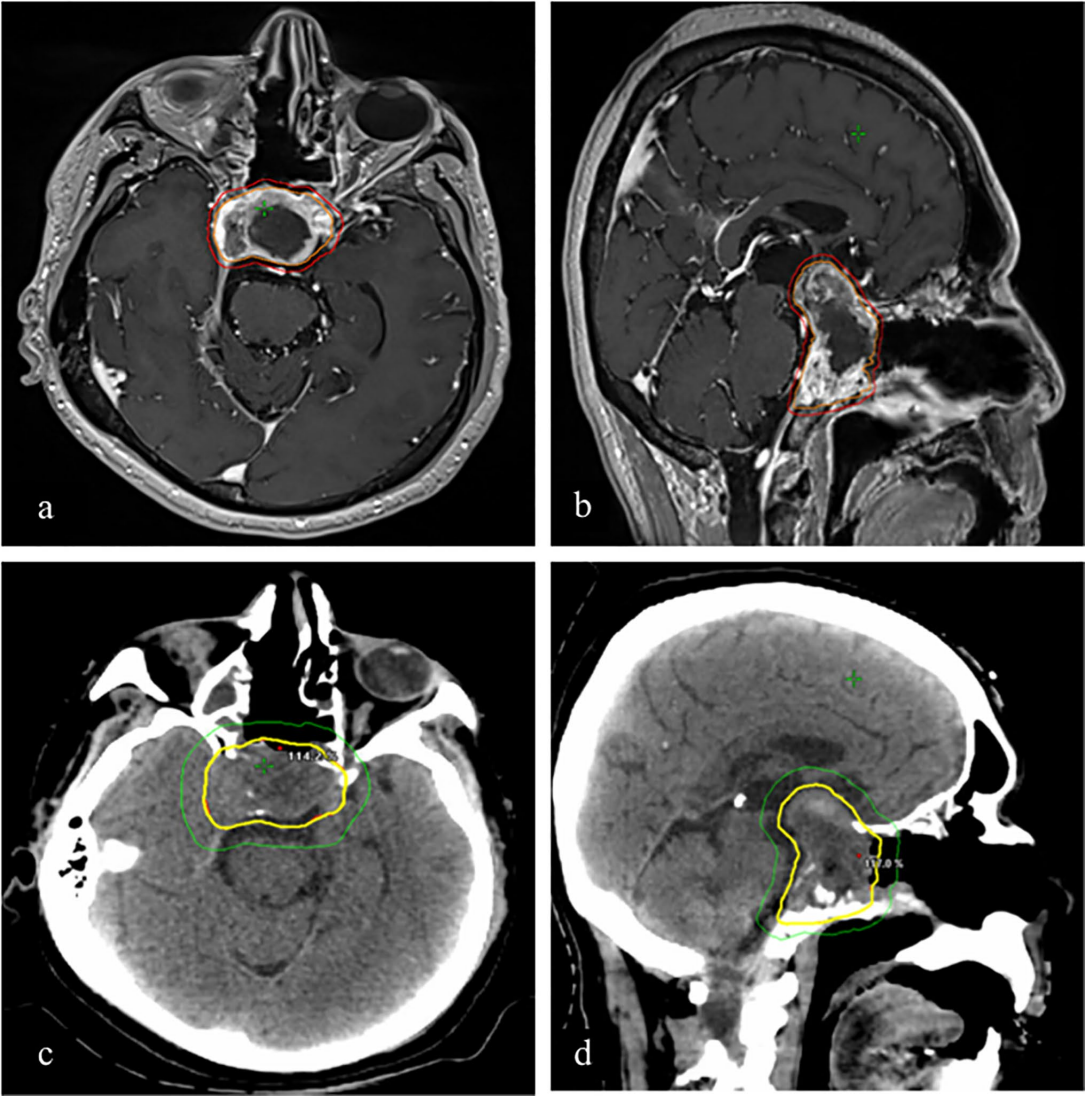
**a** & **b** Planning Target Volume (PTV) consisted of resection cavity and residual tumor identified on T1 post contrast sequence of post op MRI brain (outlined in orange) plus 2 mm margin (outlined in red). A dose of 2500 cGy in 5 fractions was prescribed to PTV by Stereotactic Radiation Therapy using 4-arc VMAT with 6MV Flatting Filter Free (FFF) photon beams to cover the PTV volume with 100% (2500 cGy) isodose line (in yellow) on planning CT head (c & d)

Subsequently, the patient received fractionated stereotactic radiation therapy to the post op bed as well as residual tumor. A dose of 2500 cGy in 5 fractions was prescribed to the planning target volume which consisted of surgical cavity and residual tumor as identified on Post op MRI with a 2 mm margin. Radiation treatment was delivered on a linear accelerator with 4-arc Volumetric Modulated Arc Therapy (VMAT) using 6MV Flatting Filter Free photon beams. Radiation plan is shown in Fig. 3.

### Case report #2

**Fig. 4:**
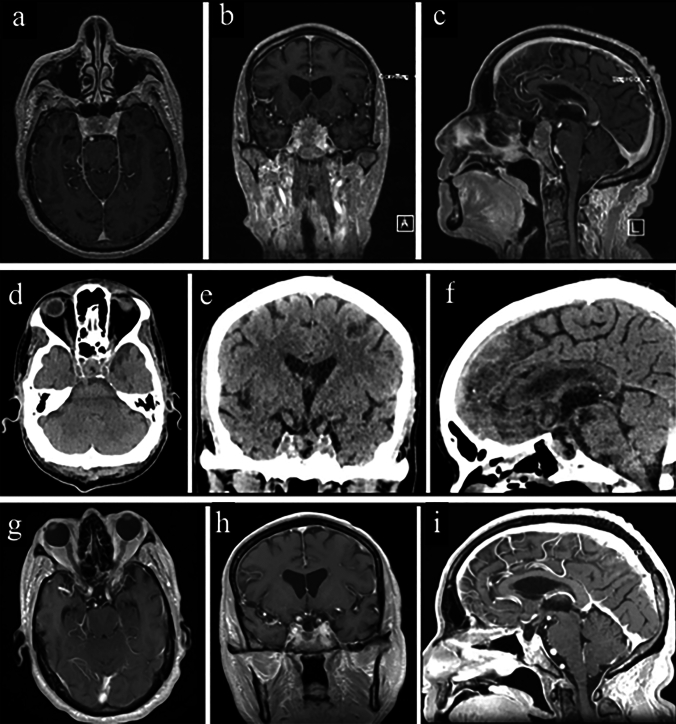
The images show radiological studies of the patient from the second case report. **a**, **b** & **c** Axial, Coronal, and Sagittal images from pre-operative brain MRI (T1-weighted post-contrast-enhanced images) revealed a heterogeneously enhancing sellar/suprasellar mass measuring approximately 26 × 16x19 mm. (**d**, **e** & **f**) Axial, Coronal, and Sagittal images from post-operative head CT show no signs of surgical site bleeding, pneumocephalus, or other complications. **g**, **h** &** i** Axial, Coronal, and Sagittal images from postoperative brain MRI (T1-weighted post-contrast-enhanced images) showing a gross total resection of the tumor

A 72 years-old male was referred to our clinic after an incidental sellar mass was discovered on imaging studies ordered for the evaluation of memory difficulties. Brain MRI revealed a heterogeneously enhancing sellar and suprasellar mass with asymmetric erosion of the sellar floor, abutting and elevating the optic apparatus. Interestingly though, the patient did not exhibit any cranial nerve deficits and pre-operative hormone levels were within normal range, indicating a non-functional PitNET. The patient underwent endoscopic endonasal transsphenoidal tumor resection without any intra or post-operative complications (Video 2) (Fig. 4).

**Fig. 5:**
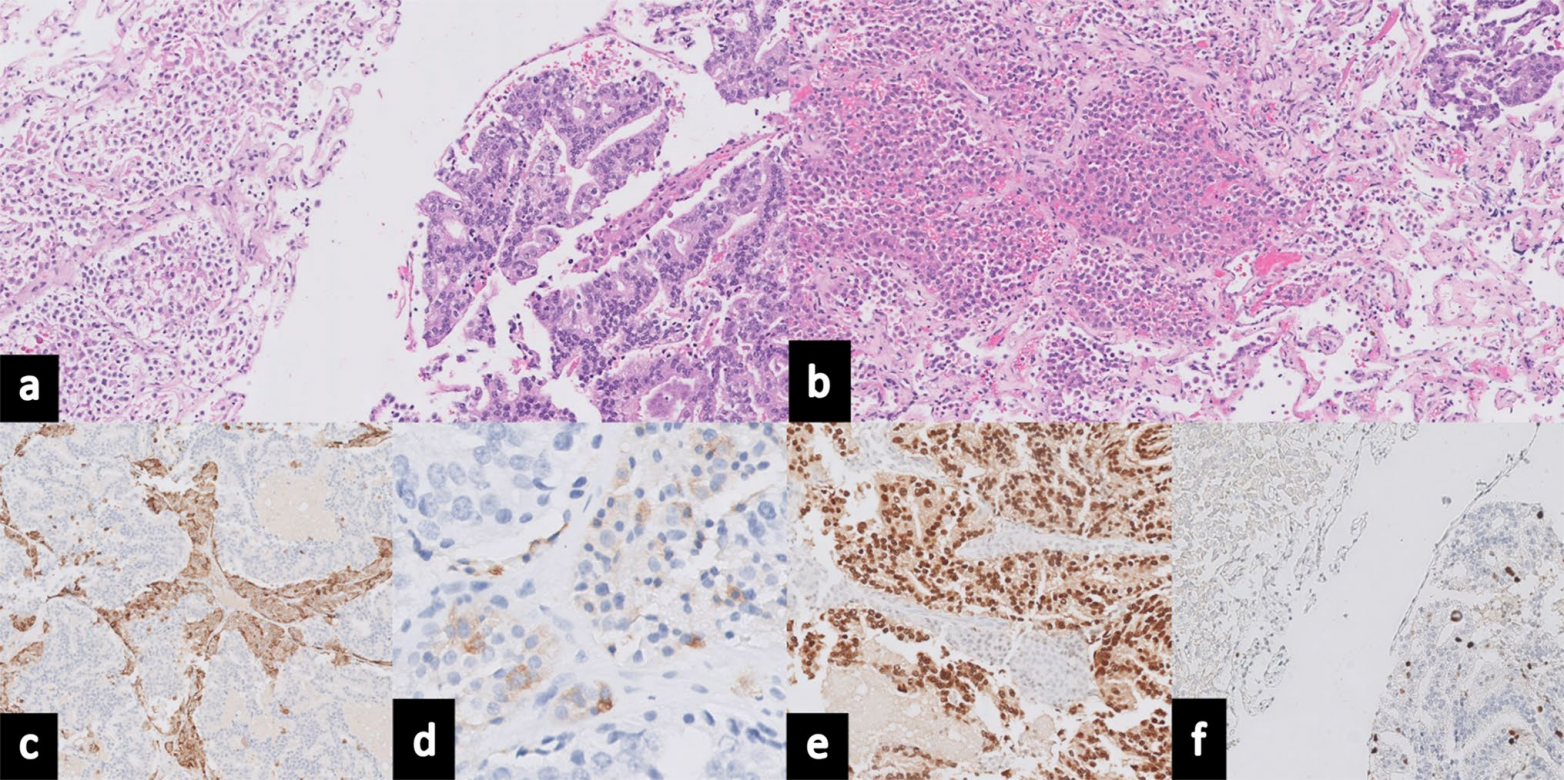
Histopathological Analysis from Surgical Resection in Case Report 2: **a** H&E staining reveals contrasting histology: the left side of the image displays characteristics of prostatic acinar adenocarcinoma, while the right side shows features consistent with PitNET. **b** Additional H&E staining depicting an intermingled pattern of the two distinct neoplasms, highlighting the juxtaposition of the prostatic acinar adenocarcinoma and PitNET. **c** Immunohistochemistry demonstrates chromogranin positivity, commonly associated with neuroendocrine differentiation. **d** Immunohistochemical staining reveals positivity for Follicle-stimulating hormone (FSH), which corroborates the diagnosis of a gonadotroph subtype within the PitNET. **e** NKX3.1 immunostaining reveals positivity, a marker typically indicative of prostatic tissue. **f** Proliferation marker Ki-67 staining is shown with a low labelling index, highlighting a proliferation rate of 1% in tumor cells

During the patient’s hospital course, he reported long-standing difficulty with urination. CT imaging showed a contrast-enhancing prostate mass, whole-body nuclear bone scan revealed diffuse osseous metastases. His prostate-specific antigen (PSA) level was markedly elevated (441 ng/mL*,* NR < 6.5 ng/mL), consistent with metastatic prostate cancer. The final pathology report on the surgical specimen confirmed concomitant Follicle stimulating hormone (FSH)-secreting PitNET and metastatic prostate adenocarcinoma (Fig. 5). Details on the pathology can be found in **Supplementary Material 1**. Three months post-surgical brain MRI revealed normal changes with no signs of residual lesions (Fig. 4).

Five weeks later, the patient underwent an oncological consultation, and was started on chemotherapy and radiotherapy for metastatic prostate carcinoma. However, his clinical course was later complicated by severe weight loss, dysphagia, anemia, acute kidney injury, metabolic acidosis, and liver failure, ultimately leading to his demise less than a year after the surgical intervention.

## Methods

### Systematic review

**Fig. 6:**
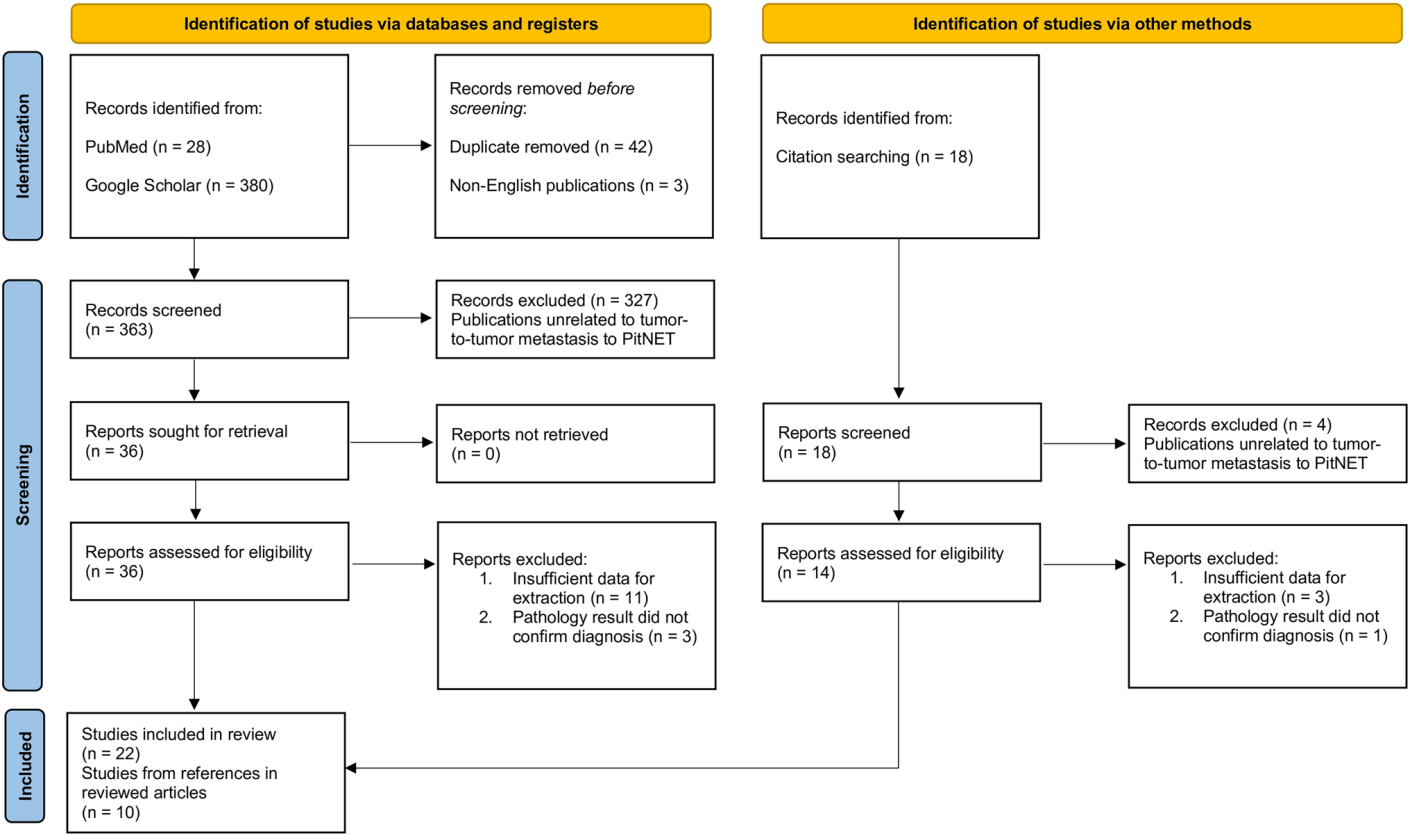
Preferred Reporting Items for Systematic Reviews and Meta-Analyses (PRISMA) flow diagram for the systematic review

A systemic review was conducted on PubMed and Google Scholar databases according to the Preferred Reporting Items for Systematic Reviews and Meta-Analyses (PRISMA) guidelines [69] (Fig. 6). The search utilized a combination of keywords that included "pituitary adenoma," "metastasis," "tumor-to-tumor metastasis," and "collision tumors". Additionally, referenced papers within identified studies were thoroughly reviewed. The search was restricted to articles published in English, with no time frame limitation. Selected publications were screened by two independent reviewers and a third reviewer adjudicated unresolved discrepancies.

### Inclusion and exclusion criteria

The inclusion criteria for this systematic review were as follows: (1) reported cases of tumor-to-tumor metastasis involving PitNETs, and (2) documentation of clinical, radiological, and histopathological findings. Studies were excluded if they did not meet these criteria or if they were review articles, case series without individual patient data, or non-English publications.

### Data extraction

Data was collected and organized into a table to facilitate analysis. Variables encompassed the primary site of the metastasizing tumor, age and gender of the patients, the type of PitNET identified, tumor size measured on the longest axis, and a variety of clinical symptoms presented by the patients. Surgical outcomes and type of surgical approach taken were also documented. In instances where surgical procedures were described in the literature only as transsphenoidal surgery (TSS), they were as such, due to the indistinguishability of whether a microscopic or endoscopic method was employed. Furthermore, gonadotrophin-producing PitNETs have been classified as non-functioning for the purposes of streamlining the presentation.

### Risk of *bias* assessment

We evaluated the risk of bias in the case reports included in this study using the Joanna Briggs Institute (JBI) Critical Appraisal Checklist for Case Reports (https://www.researchgate.net/figure/The-Joanna-BriggsInstitute-JBI-critical-appraisal-checklist-for-studies-reporting_ Fig. 2_322317583). In addition, we assessed the risk of bias in this systematic review using the ROBIS tool (Risk of Bias in Systematic Reviews) (https://abstracts.cochrane.org/introduction-robis-tool-assess-risk-bias-systematicreview).

### Statistical analysis

For variables with an approximately normal distribution, means and standard deviations are presented for the sample. Univariate associations between outcomes and the independent variables of interest were evaluated using Fisher's exact test for categorical variables. Non-parametric Kaplan–Meier estimators were calculated to estimate the survival functions for time-to-event data. Survival curves were plotted, and log-rank tests were conducted to assess the difference in survival curves between groups of interest based on the Kaplan–Meier estimates. A significance level of 5% was used to determine statistical significance for all tests. Statistical analyses were performed using JASP version 0.18.3.

## Results

### Study selection and risk of *bias*

The initial literature search yielded a total of 408 potentially relevant citations. After removing 42 duplicate records, 366 citations underwent title and abstract screening, resulting in the exclusion of 342 citations. The full texts of the remaining 22 citations were retrieved for further evaluation. Additionally, a manual search of the reference lists of these 22 citations identified 10 more potentially relevant studies. (Fig. 6) The characteristics of each case report, including the two cases presented on this study, are summarized in Supplementary Table 1.

The risk of bias assessment revealed a low risk of bias in 29 studies and a moderate risk in 3 studies (Supplementary Table 2). Given the inclusion of case reports and the small sample sizes, formal evaluations of publication bias and heterogeneity across studies were not conducted due to the inherent limitations of such analyses with this study design. However, the potential for bias within the synthesis and interpretation of findings cannot be entirely ruled out, as mentioned previously.

### Patient population characteristics

This systematic review encompassed a total of 38 cases of PitNETs harboring metastasis from various anatomical sites, including the breast (n = 7, 18.4%), colon (n = 4, 10.5%), lung (n = 8, 21.1%), and other sites such as the esophagus, stomach, pancreas, prostate, renal, mediastinum, and melanoma. The mean age of the patients at the time of metastasis diagnosis was 65 ± 11 years, with an equal gender distribution (19 males, 50%). The most common clinical presentations were visual deficits (n = 28, 74%), followed by headache (n = 8, 21%), diplopia (n = 8, 21%), fever (n = 1, 2.6%), and hemiparesis (n = 1, 2.6%). The majority of the cases involved FSH/LH-secreting PitNETs (21%, n = 8), while null cell adenomas and prolactinomas each accounted for 18% (n = 7), growth hormone (GH)-secreting PitNETs for 11% (n = 4), adrenocorticotropic hormone (ACTH)-secreting PitNETs for 8% (n = 3), and FSH-secreting PitNETs for 5% (n = 2) of the cases. In all cases where histology was not provided (18%, n = 7), the tumors were clinically non-functional, as well as in the cases with gonadotroph-secreting PitNETs. The average size of the pituitary tumor was 34.1 ± 16.2 mm in the longest axis. Patients’ demographics and data distribution are summarized in Table 1.

**Table 1:** Summary of data extracted from case reports involving malignant tumors metastasis within PitNETs

Clinical summary	
	# (%)
Age (mean ± SD)	65 ± 11
Gender	
Male	19 (50)
Female	19 (50)
Types of PitNET	
Null cell	7 (18)
Prolactinoma	7 (18)
GH	4 (11)
ACTH	3 (8)
FSH/ LH	8 (21)
FSH	2 (5)
Histology not provided	7 (18)
Clinical presentation	
Visual deficit	28 (74)
Headache	8 (21)
Diplopia	8 (21)
Fever	1 (2.6)
Hemiparesis	1 (2.6)
Type of surgery	
EEA	14 (37)
TSS	13 (34)
TC	6 (16)
NA	5 (13)
Surgical resection outcomes	
Autopsy	3 (8)
Subtotal	3 (8)
GTR	15 (39)
NA	17 (45)
Alive	
Yes	9 (24)
No	24 (63)
NA	5 (13)

*SD* Standard Deviation, *NA* Not Available, *GTR* Gross Total Resection, *EEA* Endoscopic Endonasal Approach; *TSS* Transsphenoidal, *TC* Transcranial, *GH* Growth Hormone, *ACTH* Adrenocorticotrophic Hormone

### Treatment and outcomes

Surgical resection was the primary treatment modality employed for the management of metastatic PitNETs. Endonasal endoscopic approaches (EEA) were utilized in 37% of cases (n = 14), transcranial surgeries (TC) in 16% (n = 6), and transsphenoidal surgeries (TSS) in 34% (n = 13). Subtotal resection was achieved in 39% of cases (n = 15), while gross total resection (GTR) was documented in 8% (n = 3). Notably, autopsies were performed in 8% of cases (n = 3), and surgical details were not available for 13% (n = 5) of the cases included in the analysis. Among those studies in which data was available, mean follow-up time was 10.

### Overall survival analysis

**Fig. 7:**
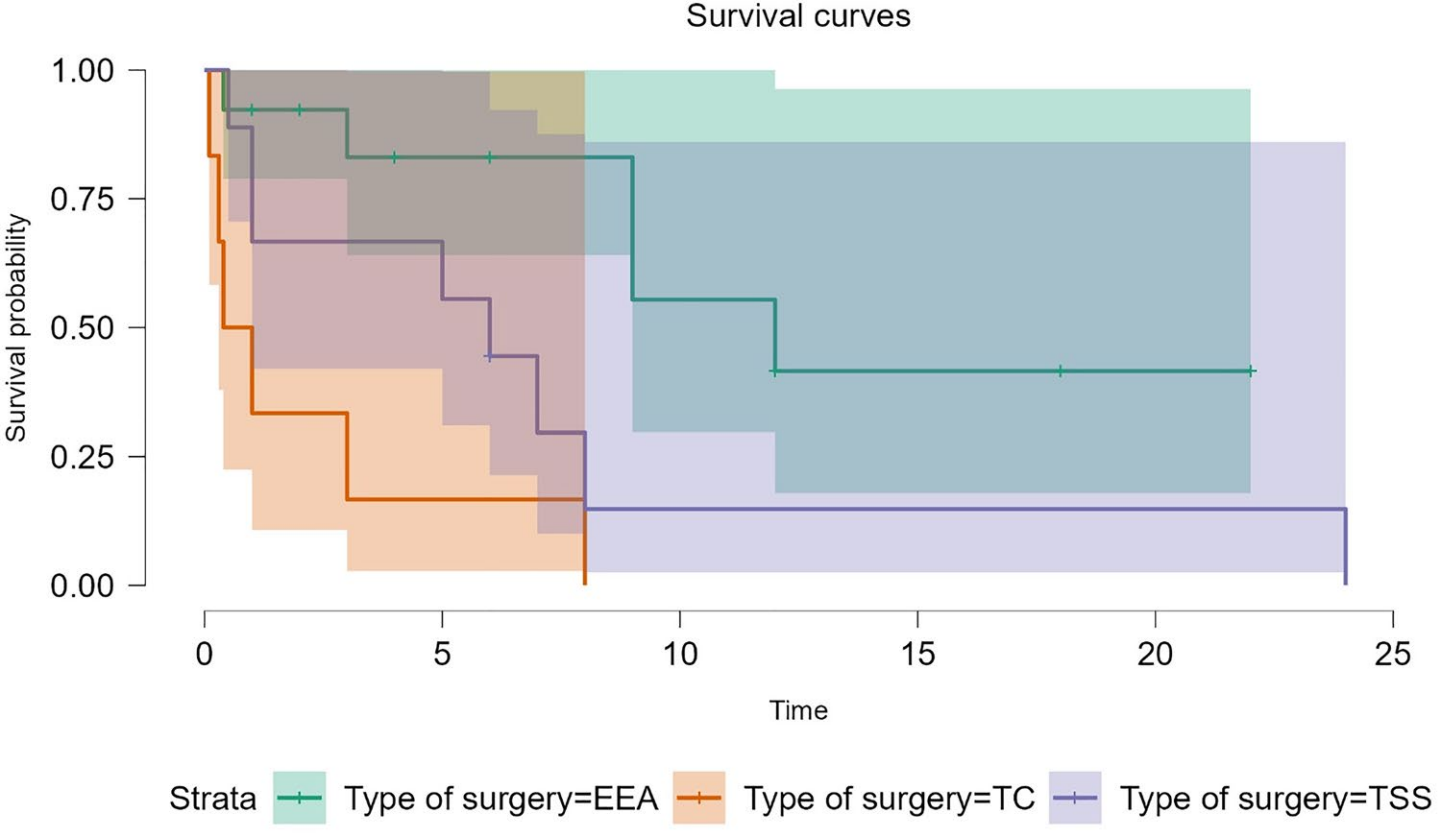
Kaplan–Meier Survival curves comparing median survival in EEA, TSS and TC approaches. EEA was found to be associated with longer median survival (p = 0.009)

The Kaplan–Meier survival analysis, which included 31 cases with available survival data, revealed a median overall survival of 7 months (95% CI: 3–12 months) in the entire cohort of patients with metastatic PitNETs. Notably, the surgical approach emerged as a significant factor influencing survival outcomes, as demonstrated by the log-rank test (p = 0.009). The choice of EEA as the surgical technique was positively correlated with improved median overall survival. Specifically, patients who underwent EEA had a favorable median survival of 12 months, compared to median survivals of 6 months for those who underwent TSS and 0.7 months for those who underwent TC. (Fig. 7).

In the overall cohort, the mean restricted survival time was 20.8 months (SE: 10.1 months). No other clinical factors, such as age, gender, tumor functional status, primary metastasis site, tumor size, or clinical presentation, were found to have a significant correlation with survival outcomes in this systematic review.

## Discussion

### General comments and comparison with related literature

The infrequency of concurrent tumors in the sellar region makes them overlooked in differential diagnoses. Clinically and radiographically, these cases usually do not present with clear signs that indicate the presence of coexisting neoplasms. However, certain clinical indicators such as rapid tumor growth and the swift symptoms progression should raise suspicions of multiple tumor pathology. As exemplified in our first case, diverse enhancement patterns on brain MRI scans may also serve as potential indicator, although such radiologic findings are not pathognomonic for tumor-to-tumor metastasis. This case also presented a substantial surgical challenge due to the lesion's high vascularity, which was likely associated with its malignant component, resulting in significant intraoperative bleeding.

Our systematic review found lung and breast cancer to be the most prevalent primary sources of metastasis to PitNETs, also known as pituitary adenomas, which is consistent with brain metastases overall [70]. The underlying mechanisms leading to the formation of metastases within PitNETs remain unclear. Some hypotheses, however, suggest that abnormalities in the pituitary gland’s vasculature, such as non-portal vessels or neovascularization in the surrounding tissues of the PitNET may provide a route for metastatic cells to invade it [64, 71].

### Clinical presentation

Visual impairments were the most frequently encountered symptom among the cases reviewed, with the severity ranging from partial loss, such as typical bitemporal hemianopsia resulting from the pressure on the fibers from nasal retina in the anterior aspect of the optic chiasm, to complete blindness, as observed in the first case we reported. The high incidence of visual symptoms correlates with the predominance of non-functioning tumors, which tend to present clinically only when their growth results in a mass effect on adjacent structures, with the optic chiasm being the most affected. The presence of malignant cells may contribute to more rapid tumor growth, potentially explaining the observed frequency of visual deficits. Furthermore, apoplexy is often observed as a clinical presentation these cases, likely due to this rapid growth, which can overwhelm the pituitary gland's venous drainage capacity. The resultant hemorrhage or infarction within the tumor leads to the sudden onset of symptoms associated with pituitary apoplexy, including severe headache, visual disturbances, and hormonal deficiencies. Headaches are believed to stem from the stretching of the dura mater as the tumor enlarges, a condition that would be anticipated with substantial suprasellar masses that exert pressure on the optic apparatus. However, headaches' more generic nature as a symptom might lead to underreporting in relation to visual disturbances.

### Surgical outcomes

The high prevalence of subtotal resection, observed in 39% of cases, reflects the aggressive nature and complexity of those cases. We did not find this high rate to be explained by the used surgical approach or technique. Beside the hypervascularity challenge that was overmentioned, the rapid growth of metastatic cancer disrupts normal anatomical planes. This increases adherence to neurovascular structures, making total resection extremely challenging. On top of that, only 24% of patients remained alive on the latest follow-up, which further emphasizes the severity of this condition.

### Strengths and limitations

Our review highlights that the choice of endoscopic approaches over other modalities has been proven beneficial to patients with PitNETs, being associated with less postoperative endocrinological abnormalities, such as diabetes insipidus and hypothyroidism, when compared to the microscopic TSS approach [72]. Mortality rates have also been observed to be higher in cases where a transcranial approach was employed compared to both microscopic TSS and EEA [73]. The finding of a longer median survival in those patients who underwent EEA in our review helps to further solidify the endoscopic approach as the standard of care, although the small number of cases and specific patient subset limit the generalizability of this data for PitNET surgeries overall. Furthermore, as another limitation, the association between the transsphenoidal approach and better outcomes observed in our study may be confounded by a selection bias, where surgeons preferentially use the transsphenoidal approach for less complex cases and reserve the transcranial approach for more challenging cases, potentially skewing the effectiveness comparison.

## Conclusion

Tumor-to-tumor metastasis to PitNETs, also known as pituitary adenomas, presents a unique intersection of oncological and endocrinological challenges, characterized by its rarity, diverse origins, and the complexity of surgical management. This study contributes to the growing body of evidence on the subject and the presented cases underscore the importance of multidisciplinary teamwork, including oncological, neurosurgical, and endocrinological, in devising the best plan to optimize provided management quality and outcomes.

## Supplementary Information

Below is the link to the electronic supplementary material.

Supplementary file1 (DOCX 12740 KB)

Supplementary file2 (DOCX 31 KB)

Supplementary file3 (DOCX 28 KB)

Supplementary file4 (MP4 645947 KB)

Supplementary file5 (MOV 1851185 KB)

## Data Availability

No datasets were generated or analysed during the current study.
