# Supplementary material for: “Intrasellar tumor-to-tumor metastasis: A single center experience with a systematic review”
Source: Pituitary. 2024 Aug 14;27(5):455–67. doi: 10.1007/s11102-024-01441-9 (PMC11513765; doi:10.1007/s11102-024-01441-9)
Supplement: Supplementary file 1 — Supplementary file1 (DOCX 12740 KB) [file 11102_2024_1441_MOESM1_ESM.docx]

**Supplementary Material – Pathology Report**

- **Case Report 1**

***Diagnosis:***

Pituitary gland, sellar-suprasellar tumor, endoscopic endonasal approach resection:

- Metastatic renal cell carcinoma, juxtaposed to gonadotroph-type pituitary adenoma.

***Comments:***

The renal cell carcinoma is of the clear cell type. No sarcomatoid features are identified. By immunohistochemistry (B2) the tumor cells are positive for AE1/3 and PAX-8.

The pituitary adenoma consists of an epithelioid neoplasm, growing in expanded lobules. Tumor cells have round to ovoid nuclei, small but distinct nucleoli, and abundant and granular eosinophilic cytoplasm. Mitotic figures are inconspicuous (<1 per 10 HPFs). Areas of infarction are present (pituitary apoplexy). By immunohistochemistry (B2) the tumor cells are positive for SF-1 and are negative for PIT-1 and T-PIT. A Ki-67 proliferation index highlights 1% of tumor cells.

***
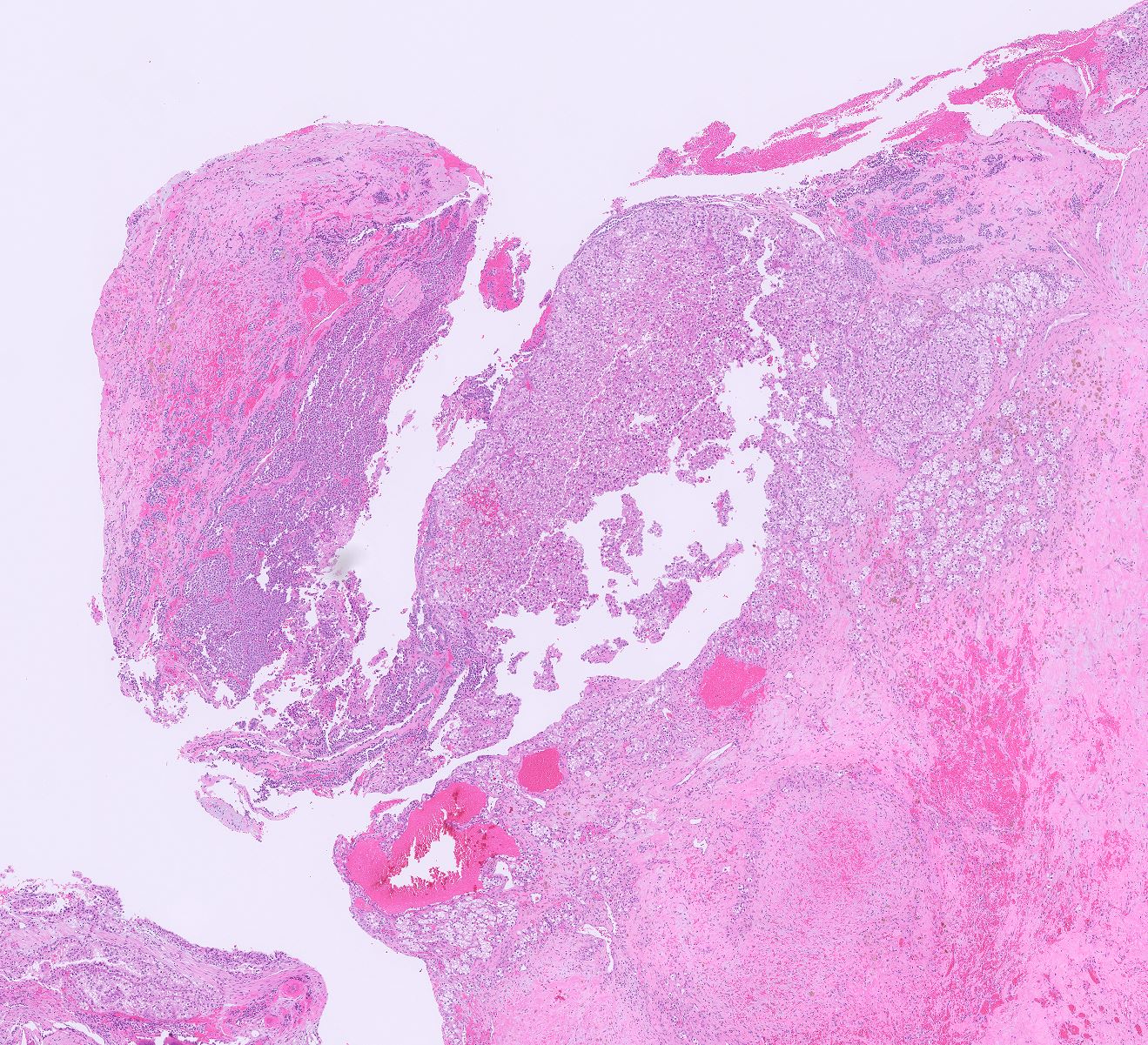
***

***Hematoxylin and Eosin, 2X***

***
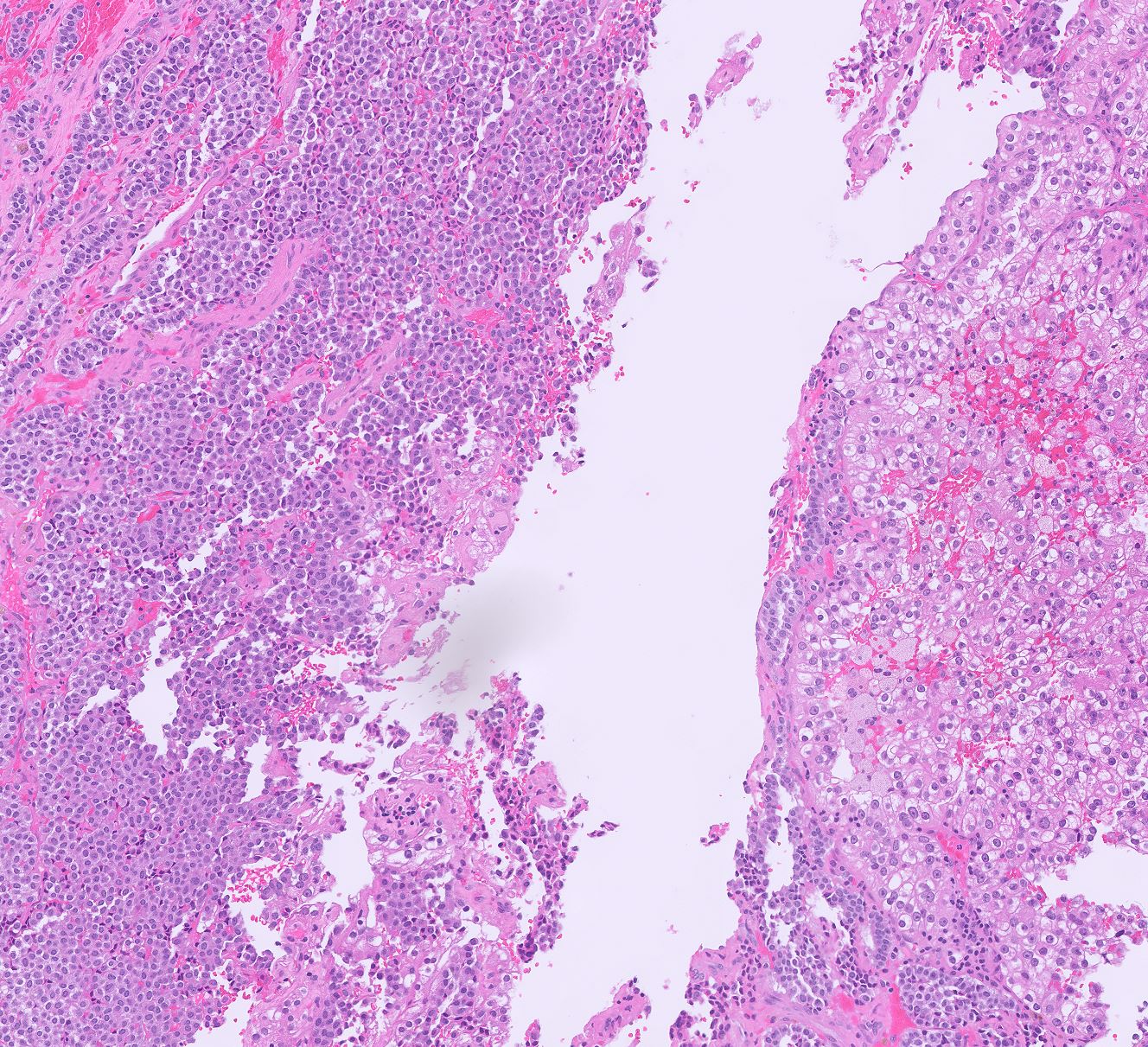
***

***Hematoxylin and Eosin, 10X***

***
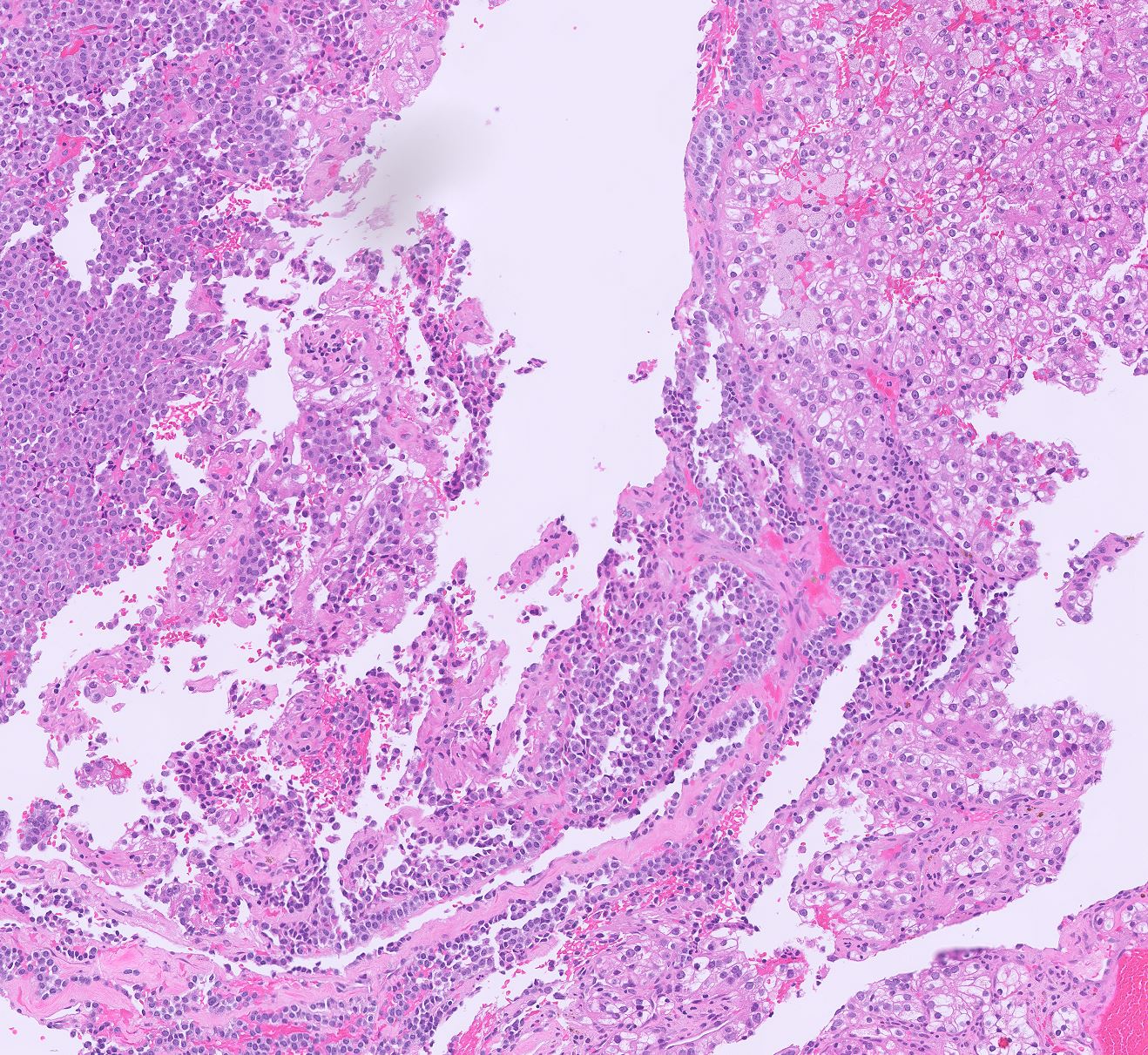
***

***Hematoxylin and Eosin, 10X***

***
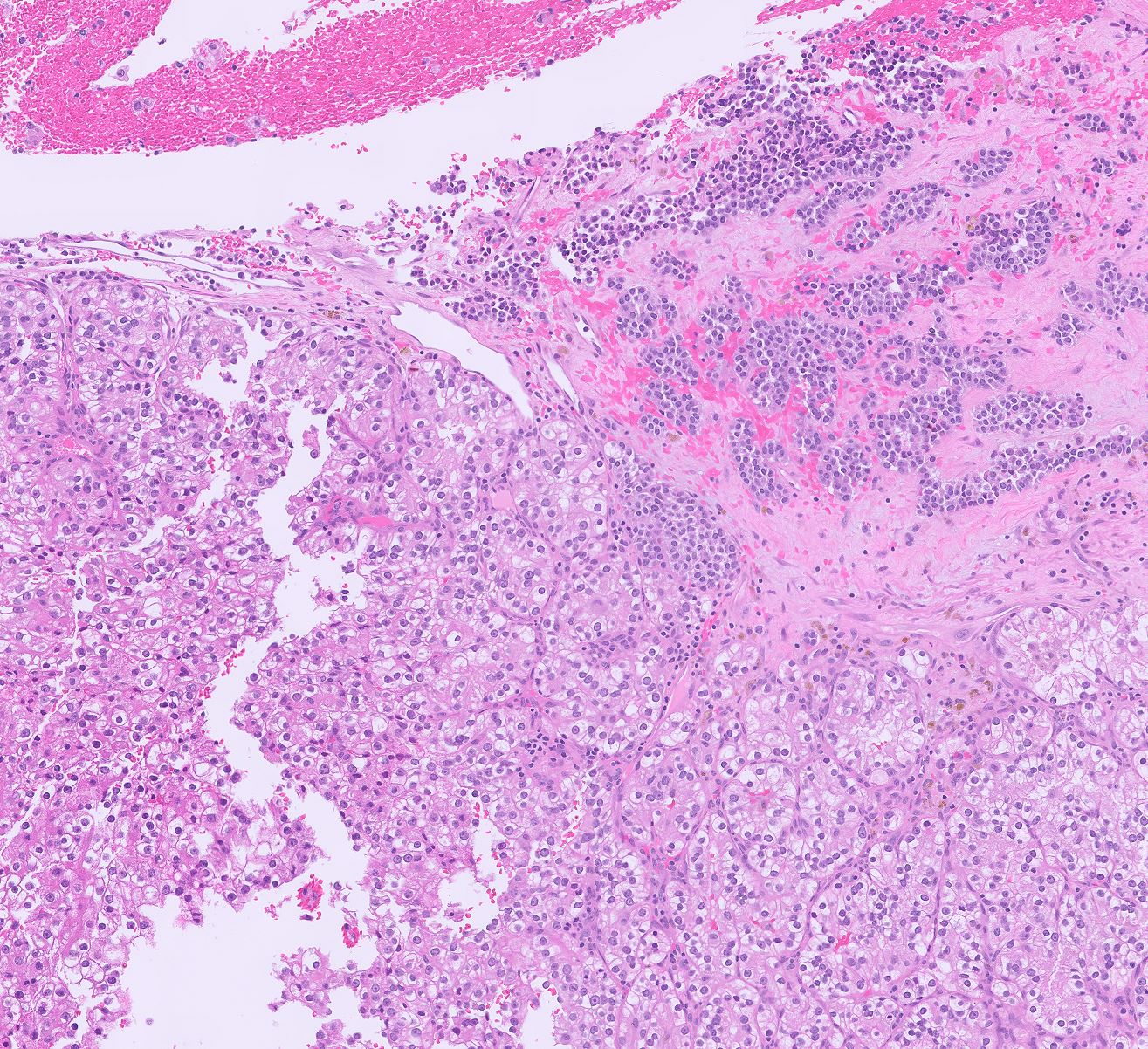
***

***Hematoxylin and Eosin, 10X***

***
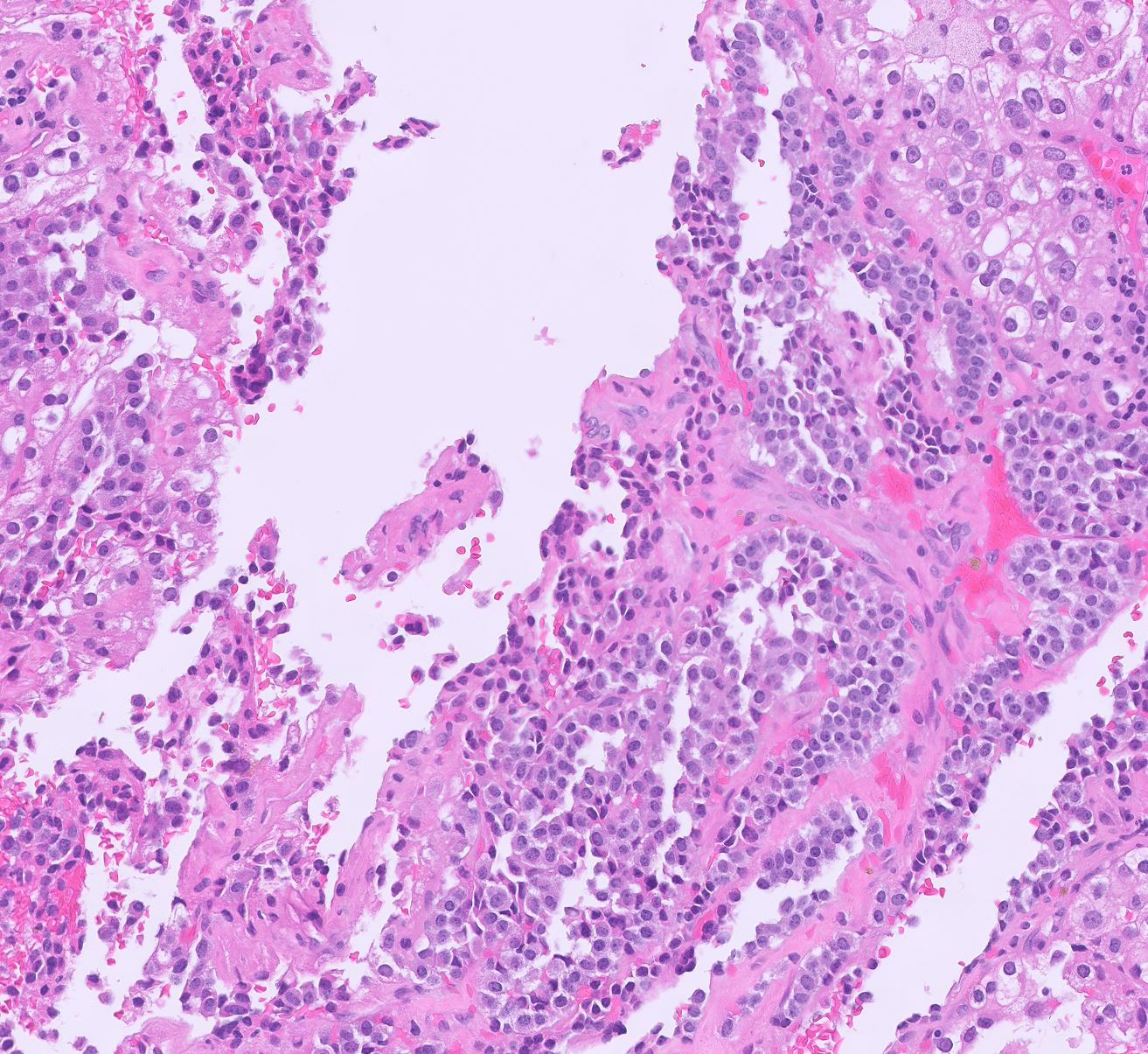
***

***Hematoxylin and Eosin, 20X***

***
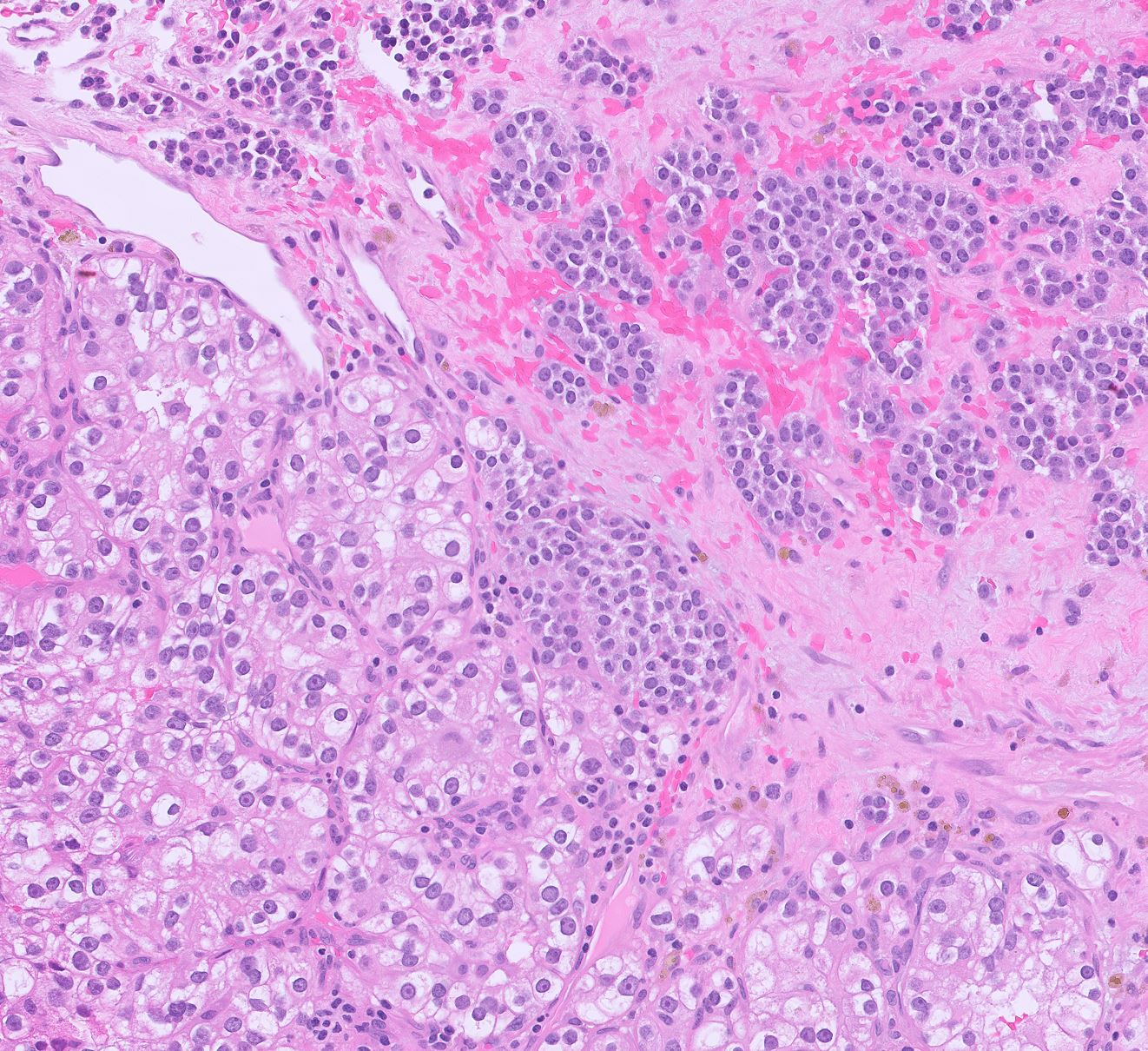
***

***Hematoxylin and Eosin, 20X***

***
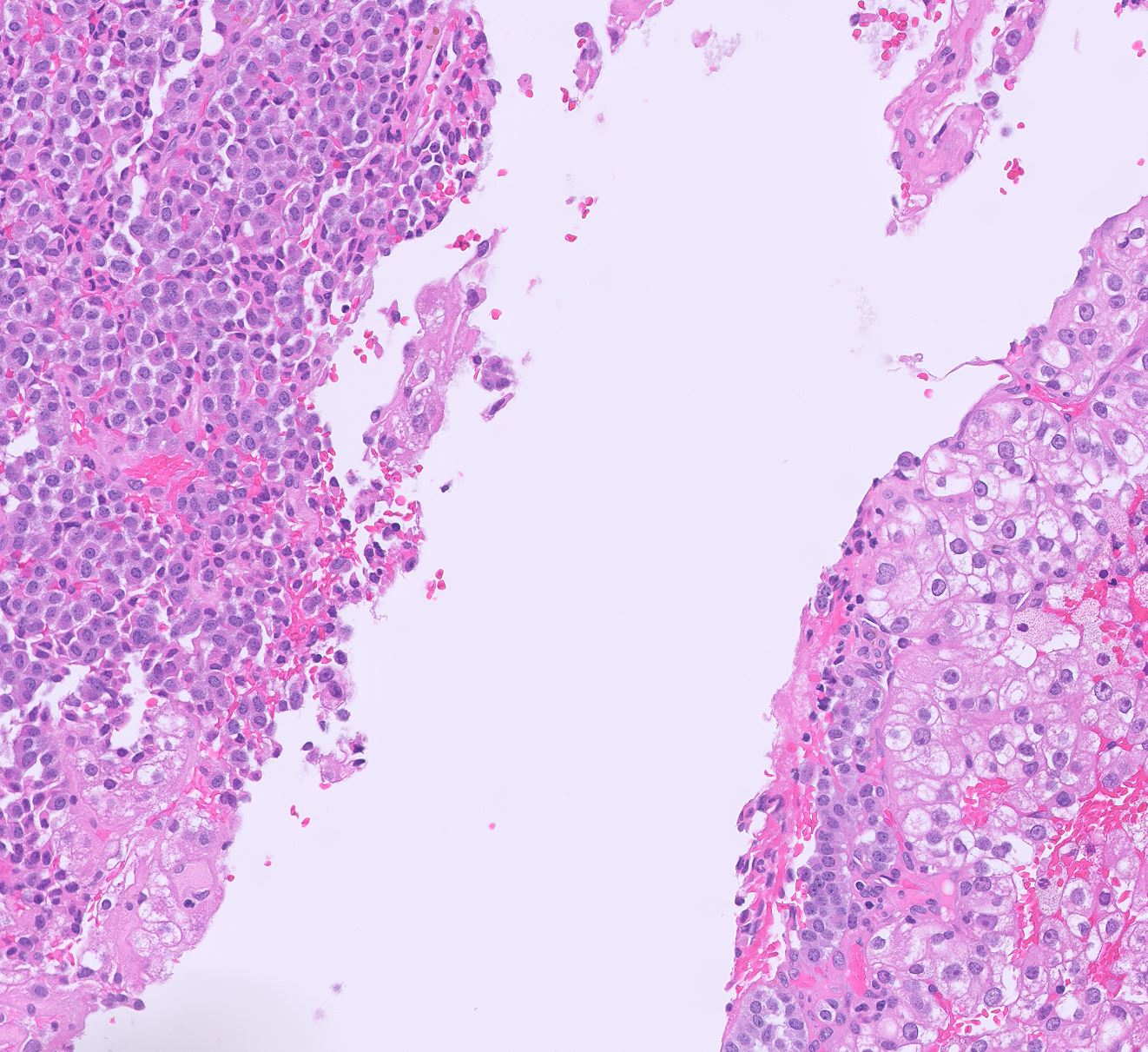
***

***Hematoxylin and Eosin, 20X***


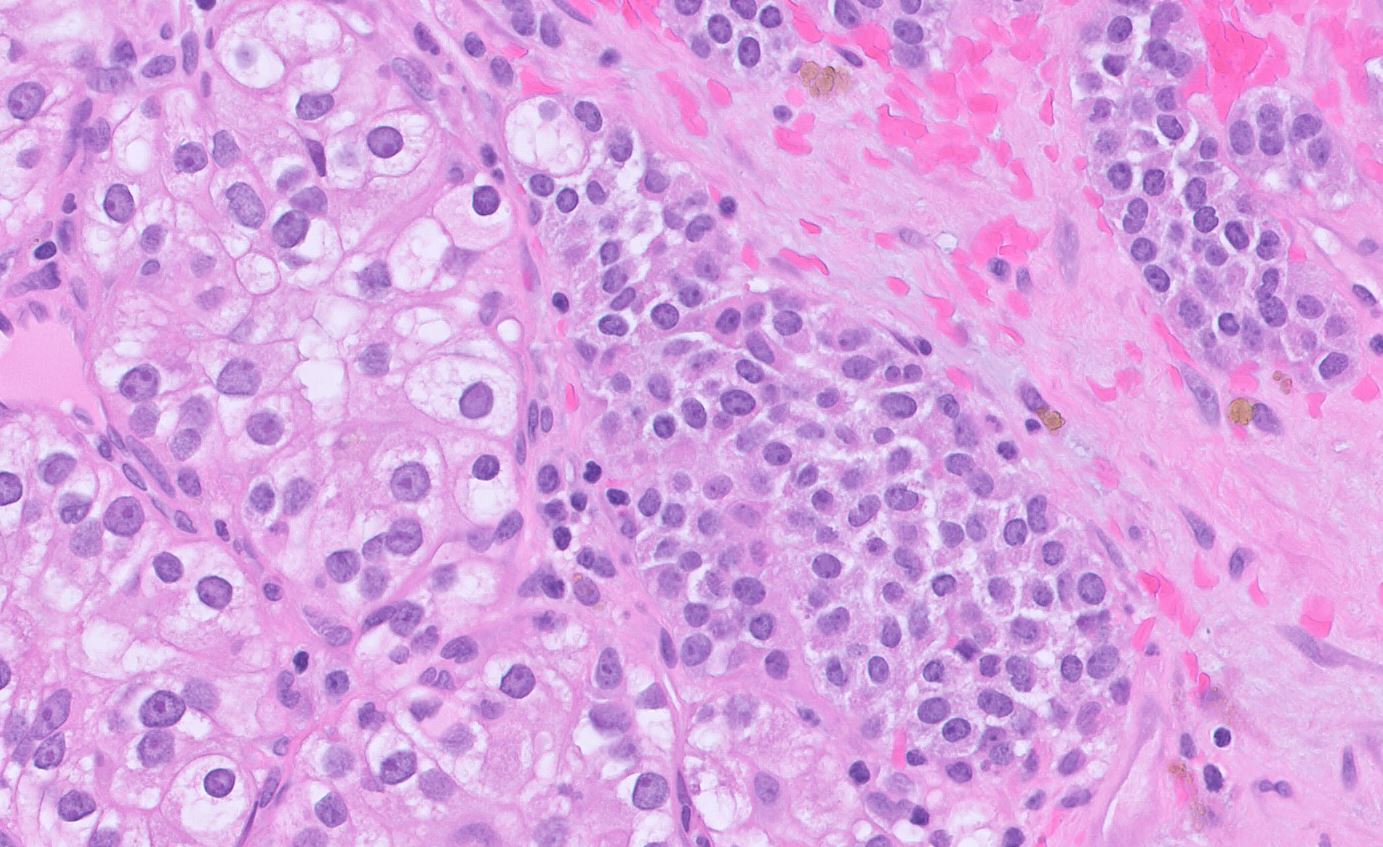


***Hematoxylin and Eosin, 40X***


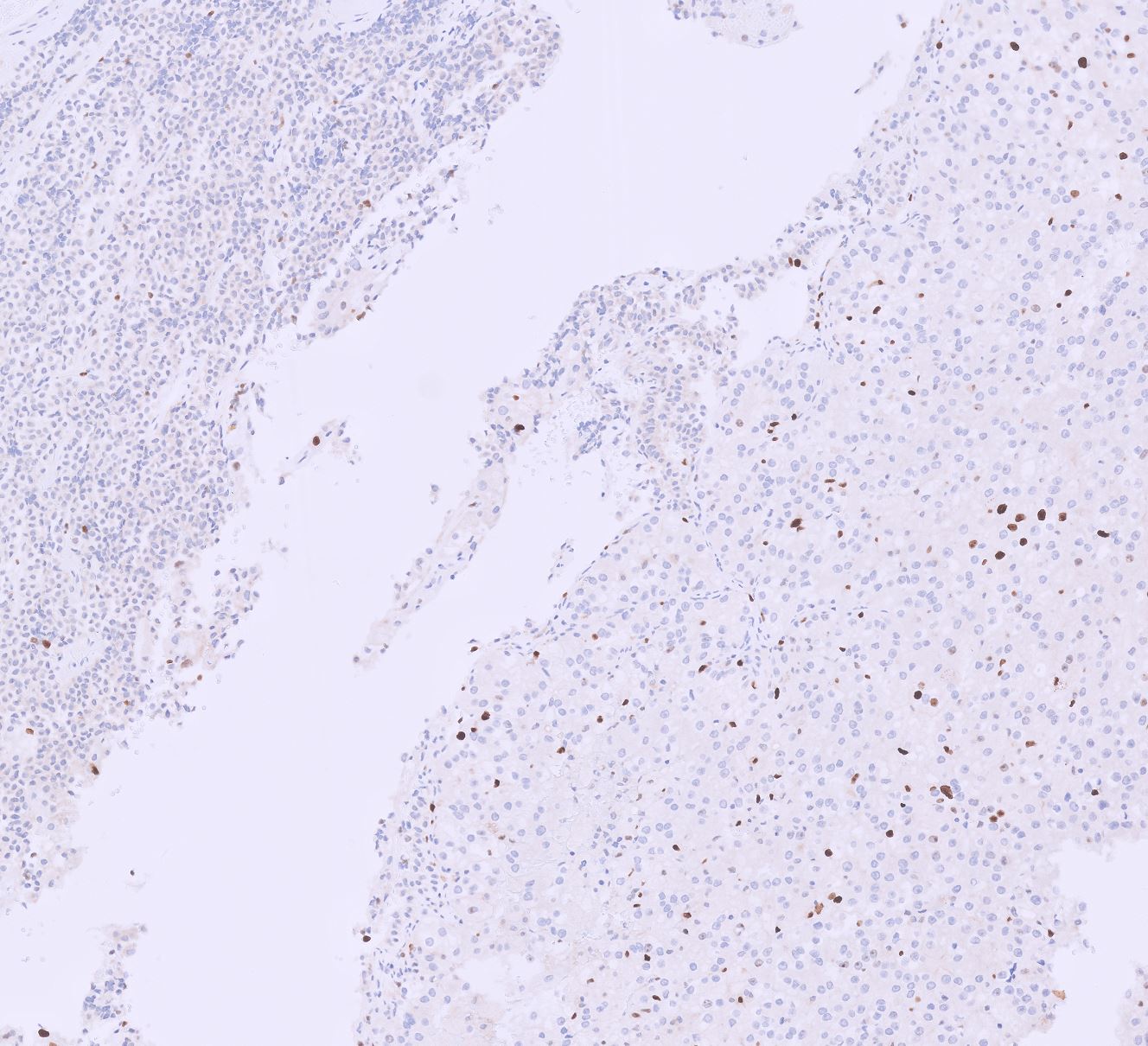


***Ki-67, 10X***


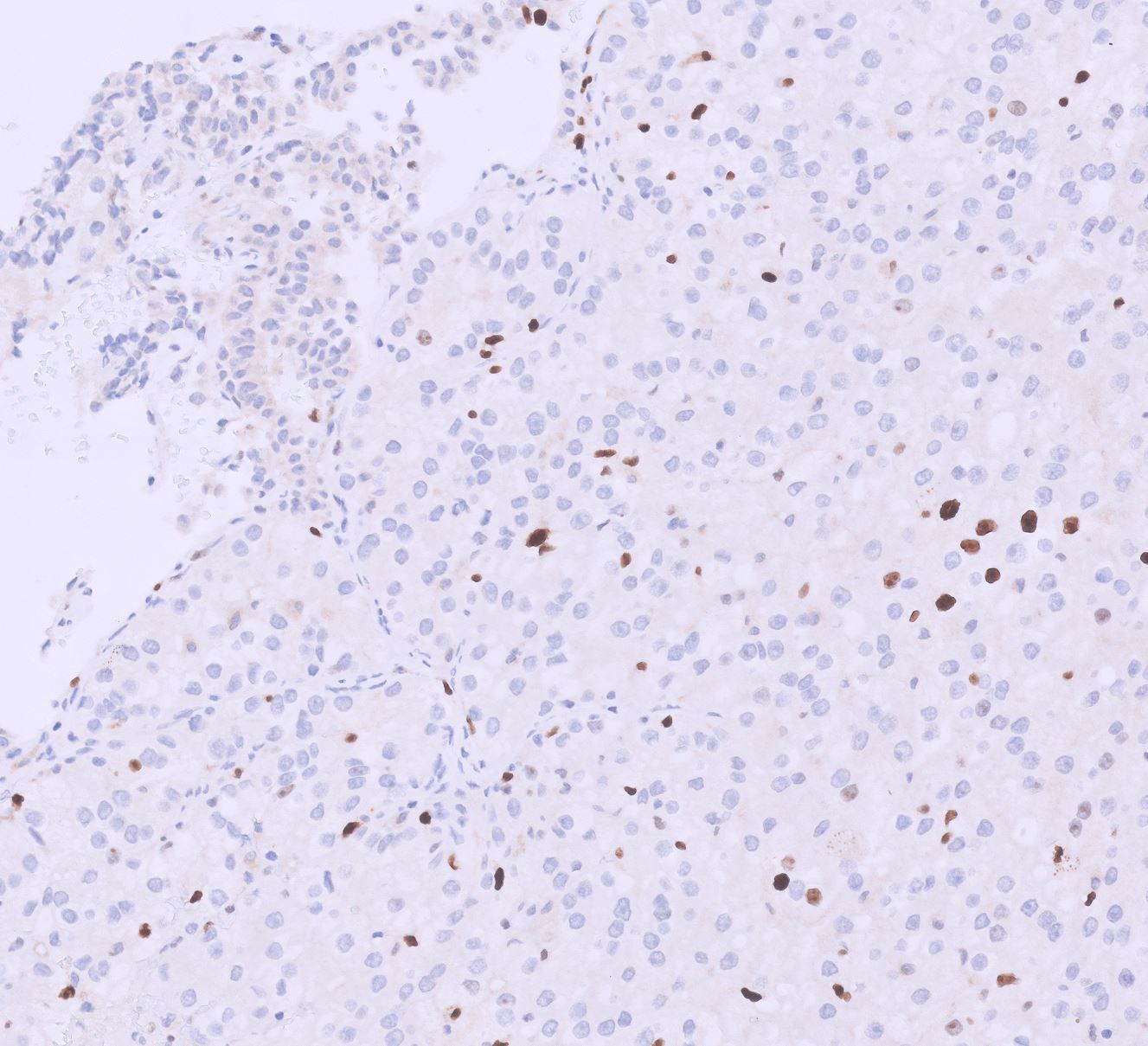


***Ki-67, 20X***

***
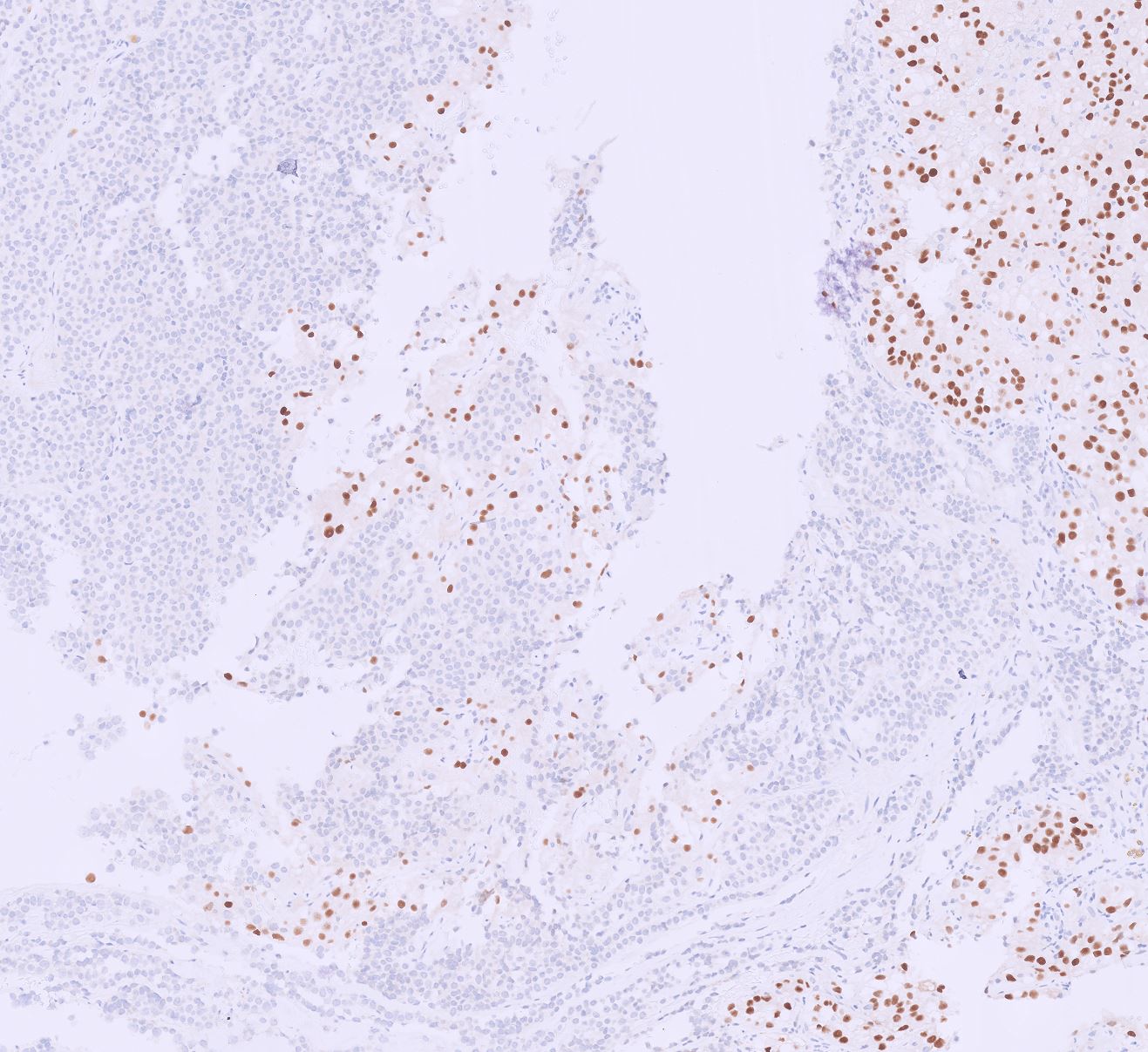
***

***PAX-8, 10X***

***
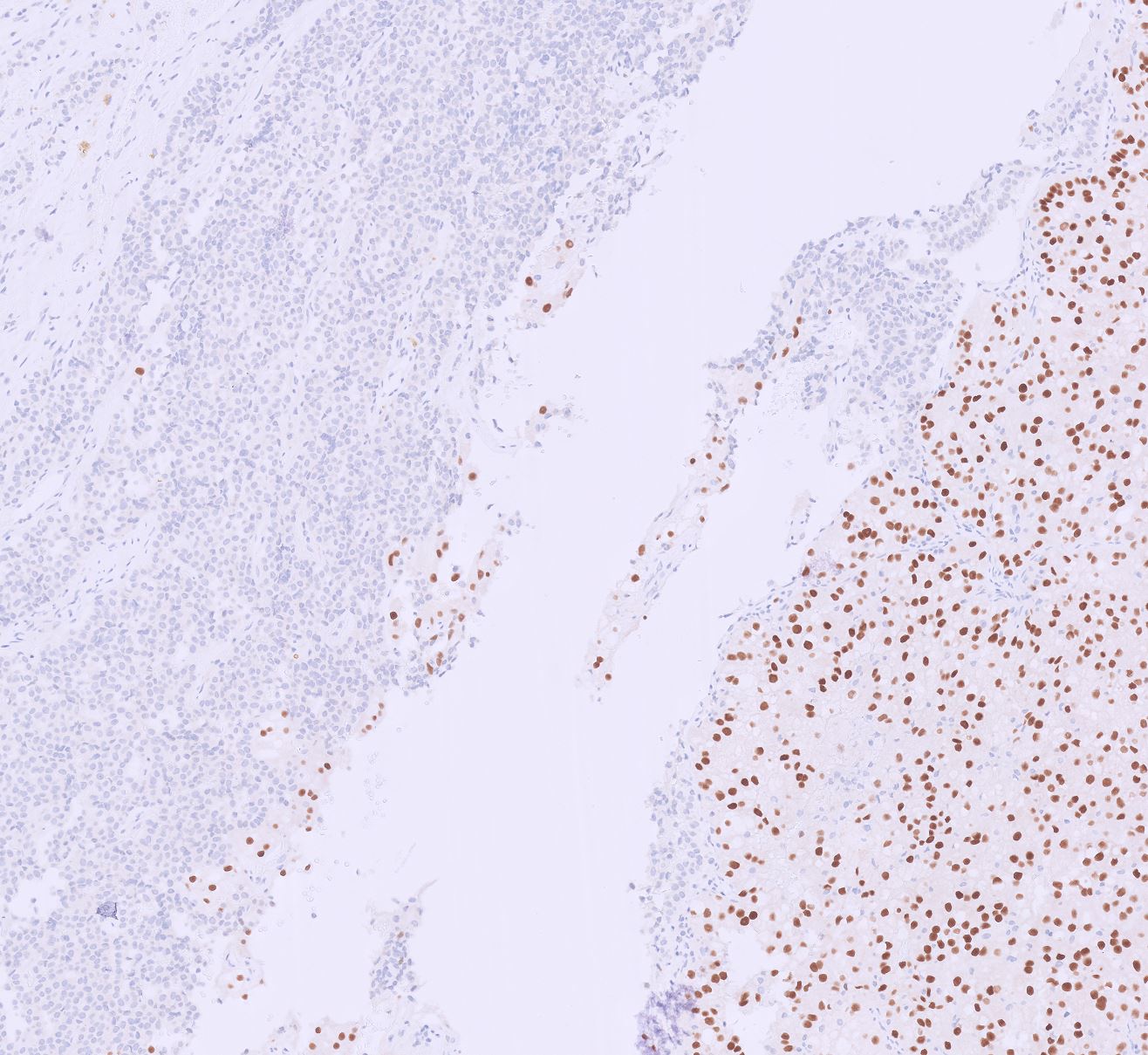
***

***PAX-8, 10X***

***
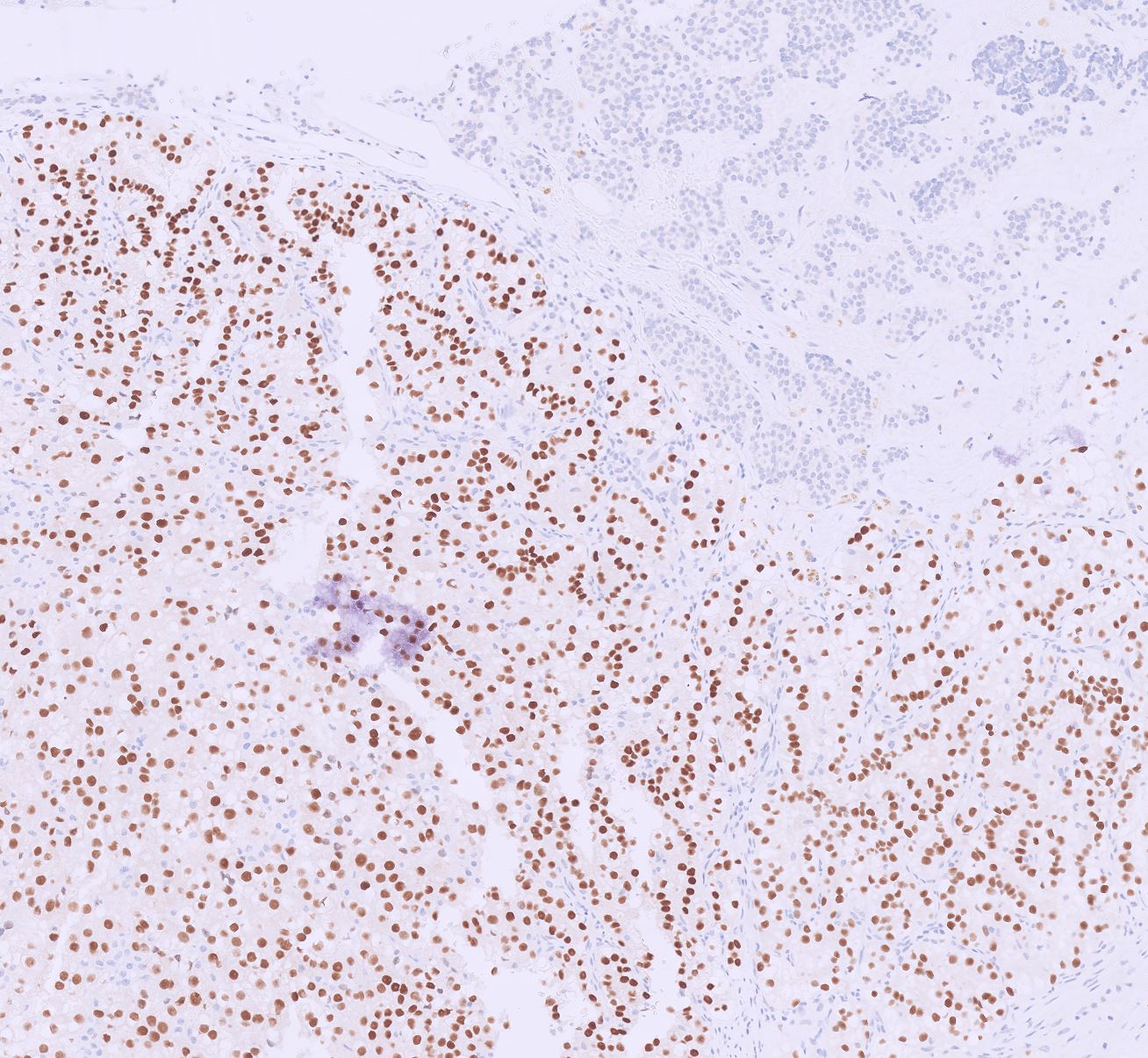
***

***PAX-8, 10X***

***
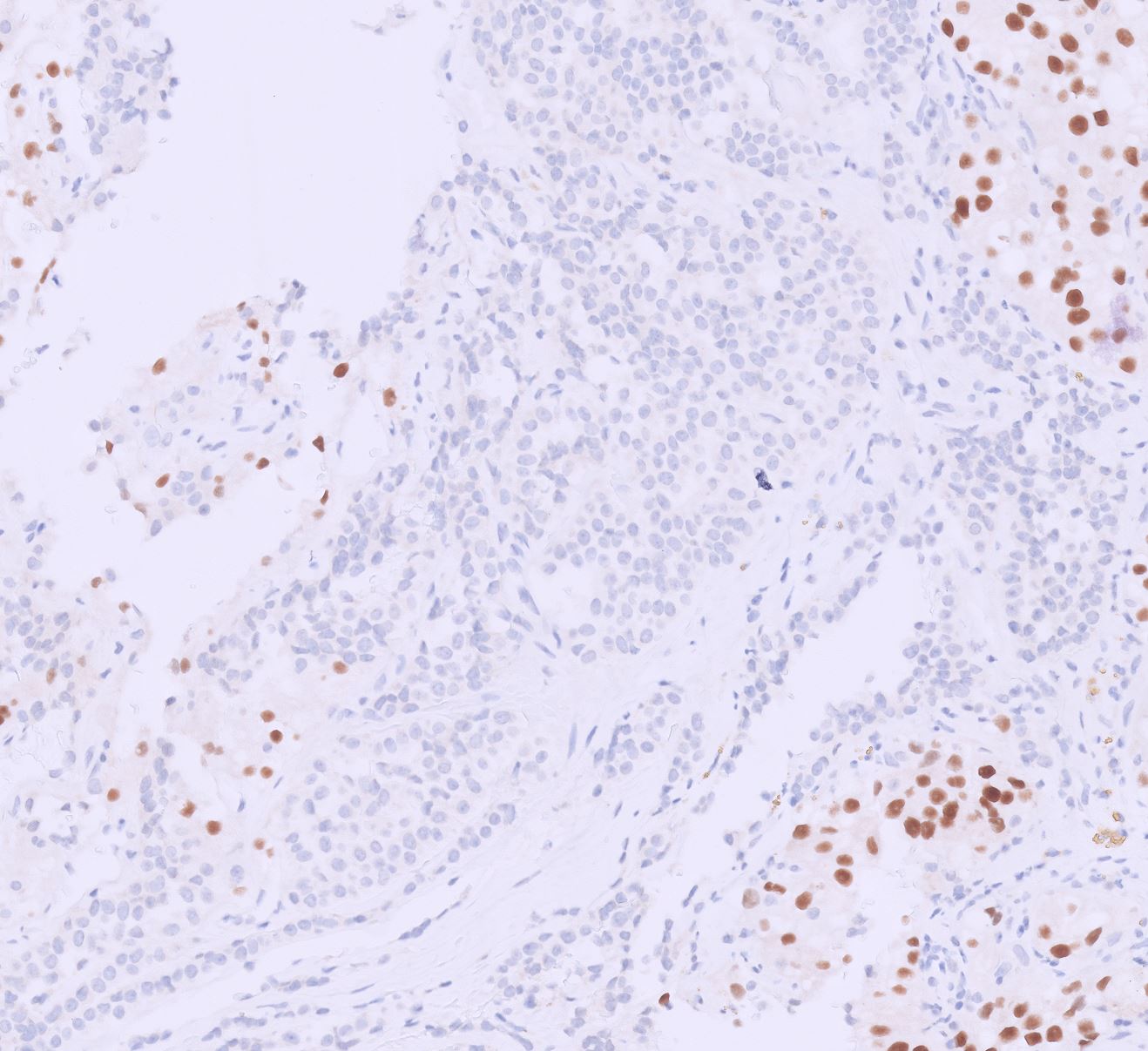
***

***PAX-8, 20X***

***
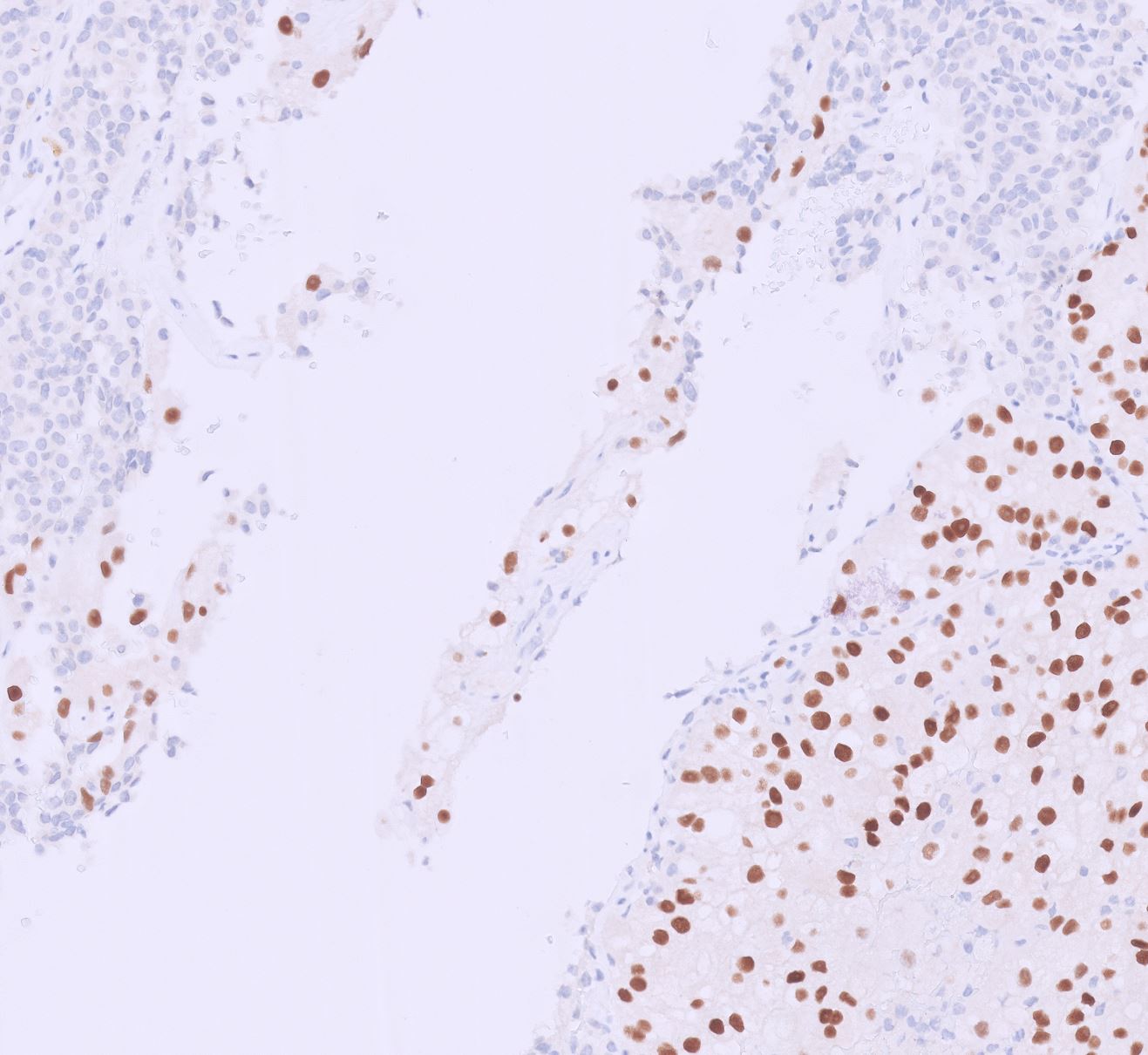
***

***PAX-8, 20X***

***
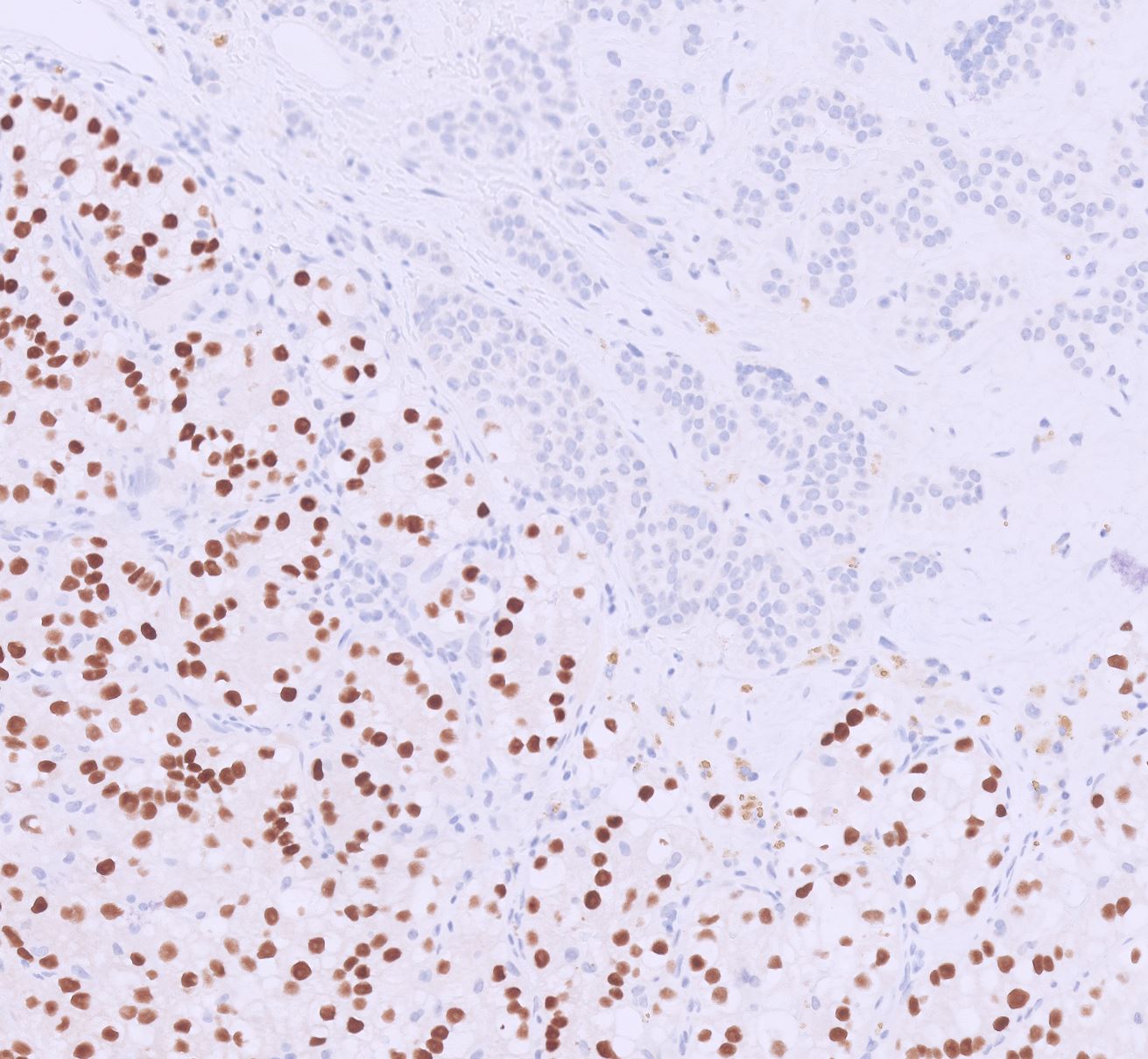
***

***PAX-8, 20X***


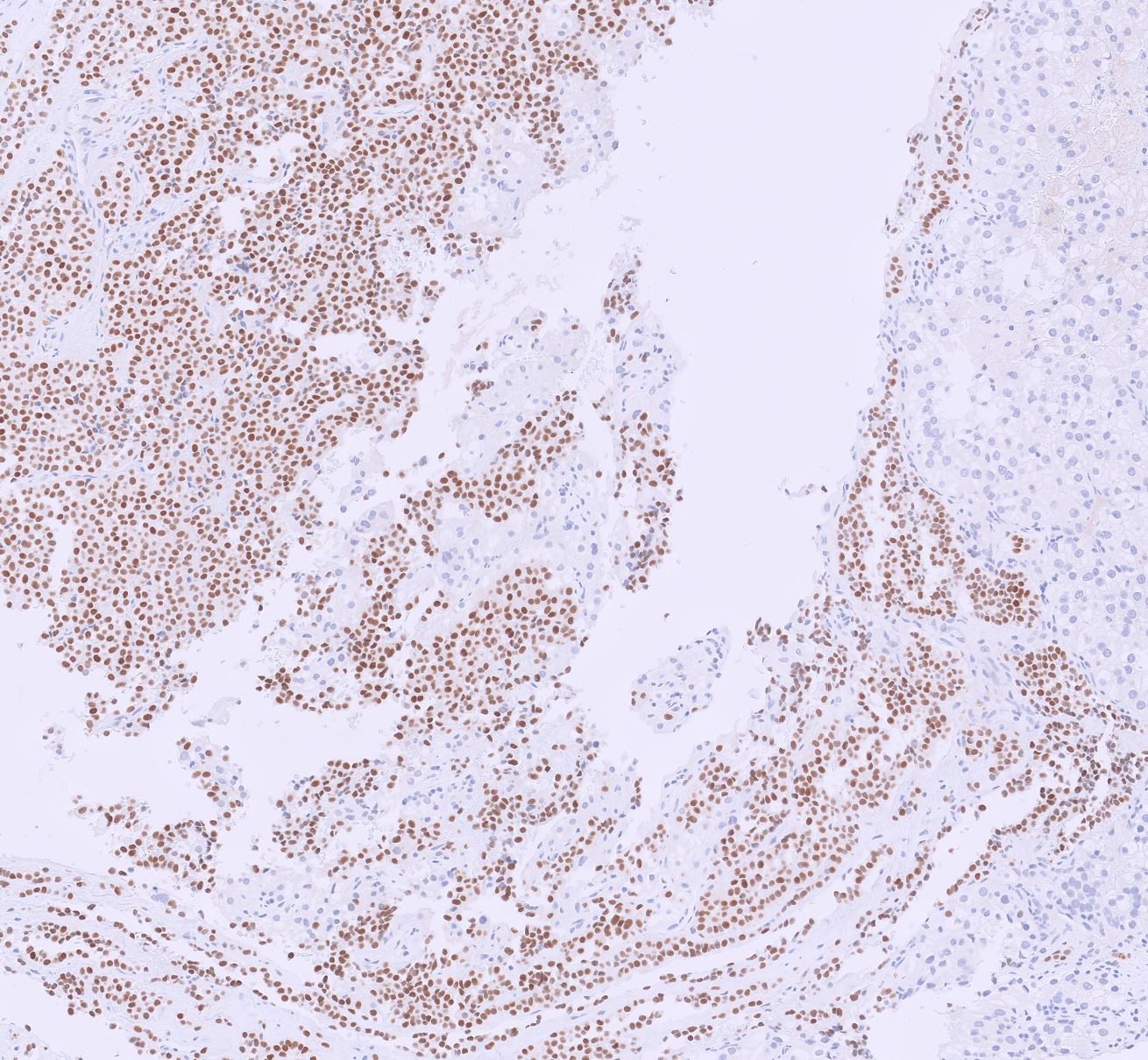


***SF-1, 10X***


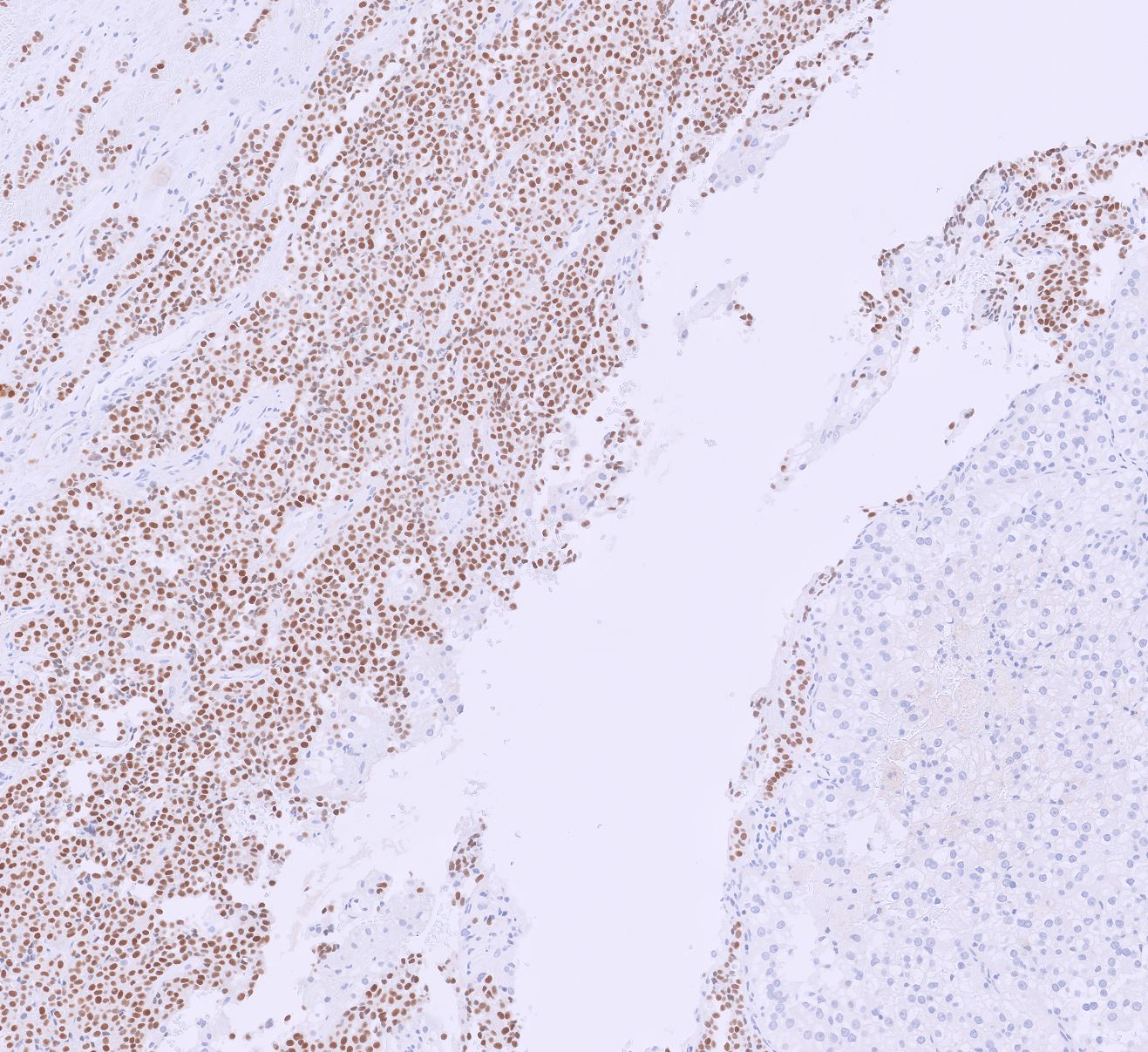


***SF-1, 10X***


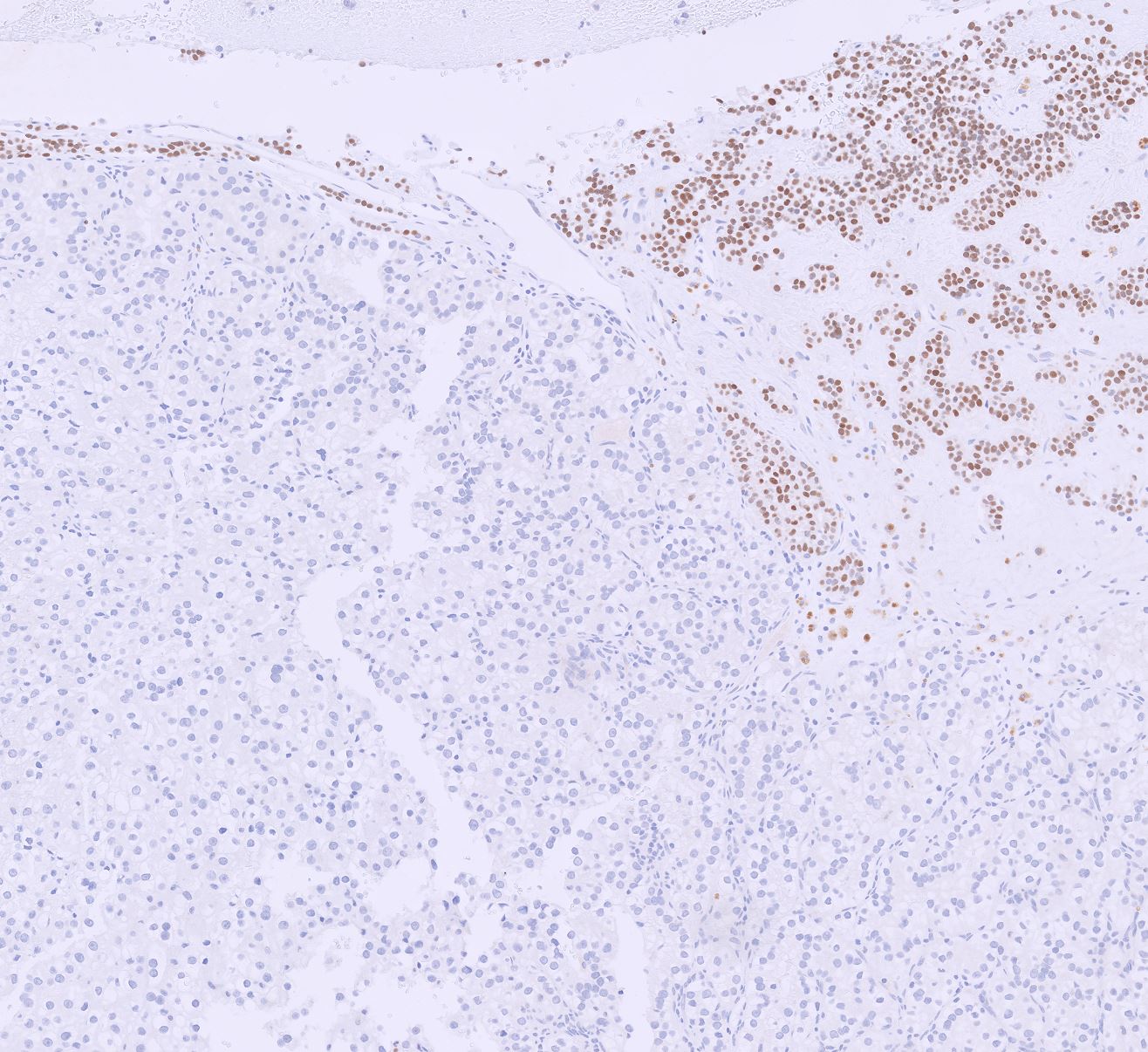


***SF-1, 10X***


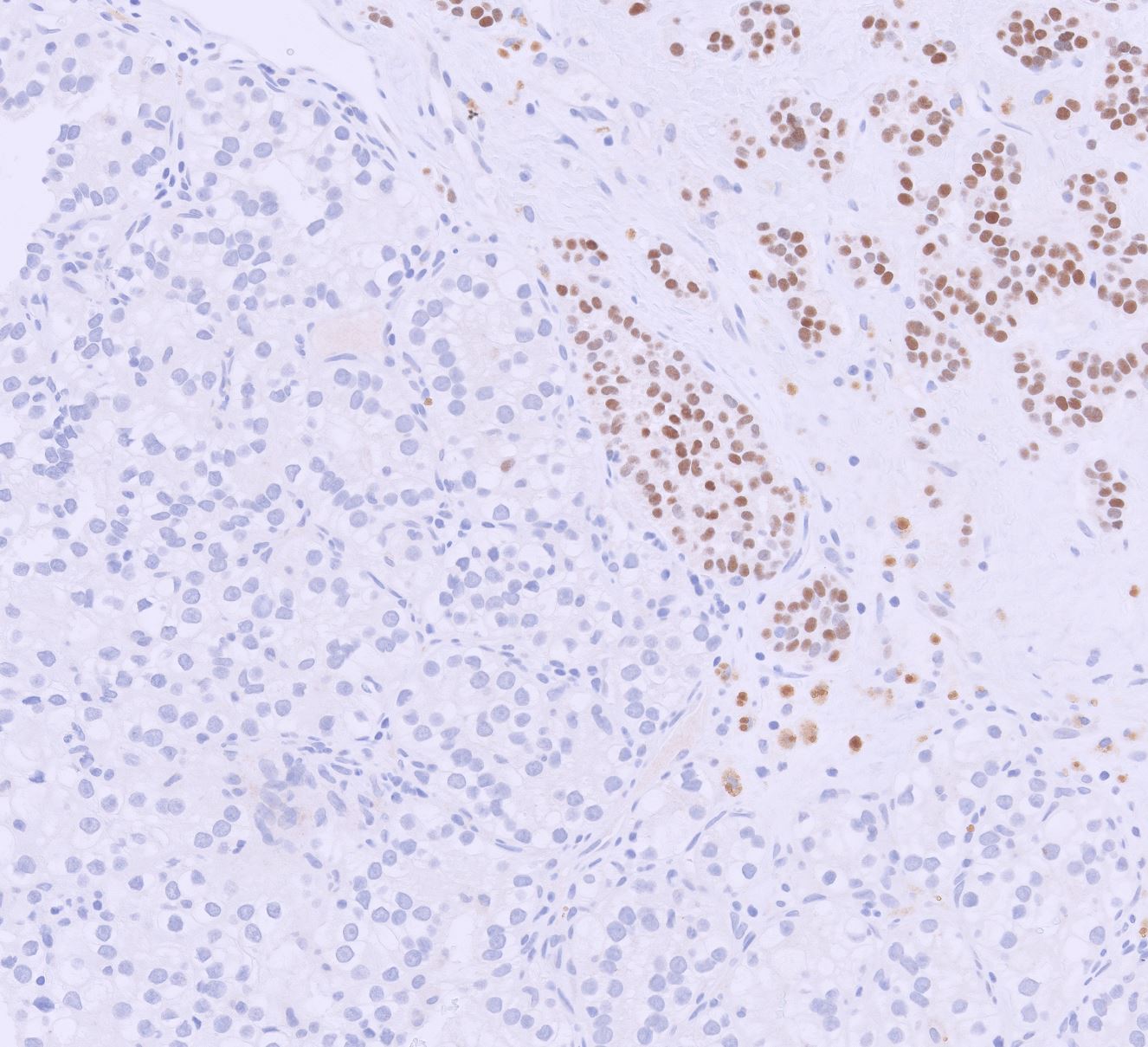


***SF-1, 20X***


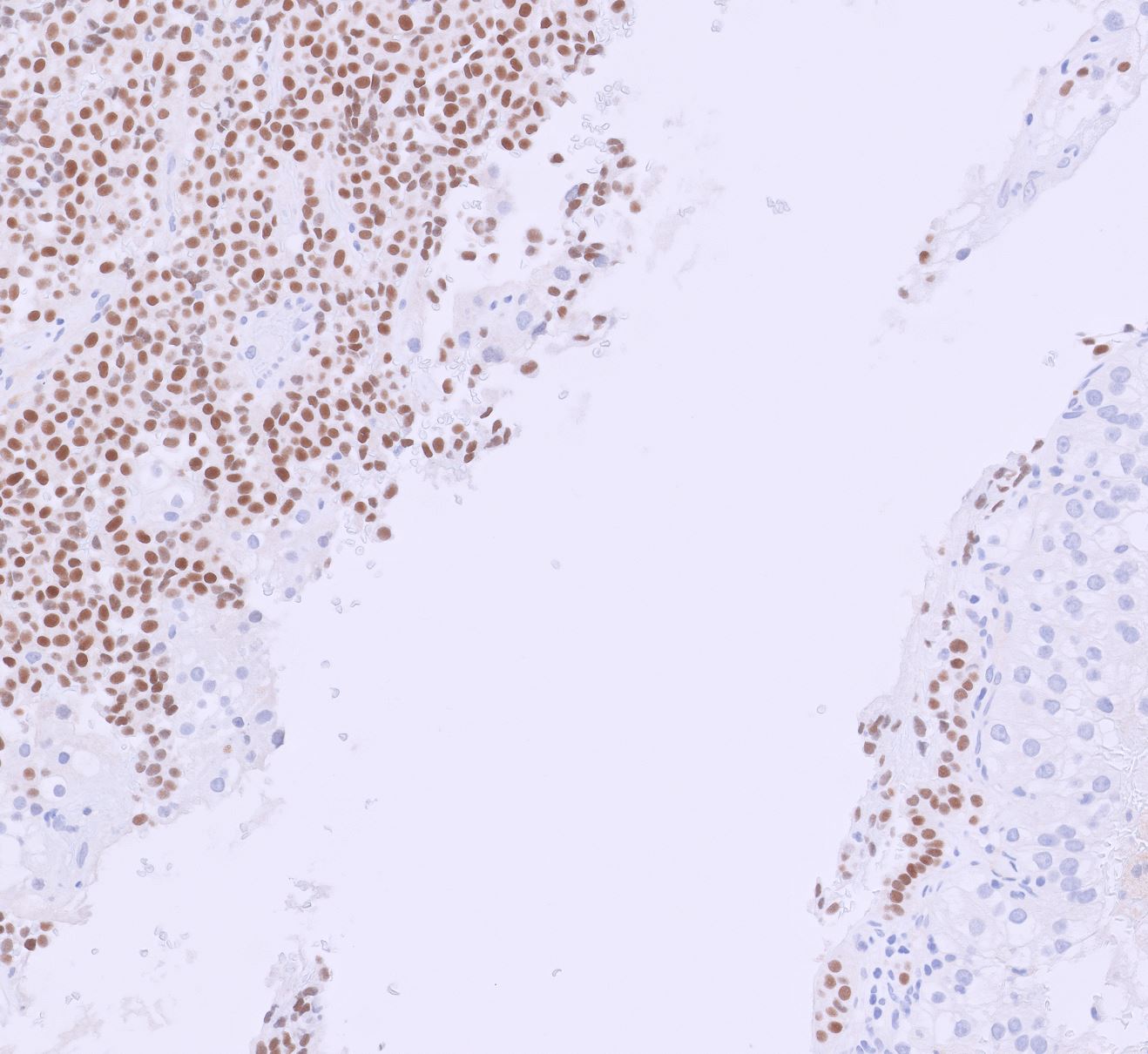


***SF-1, 20X***


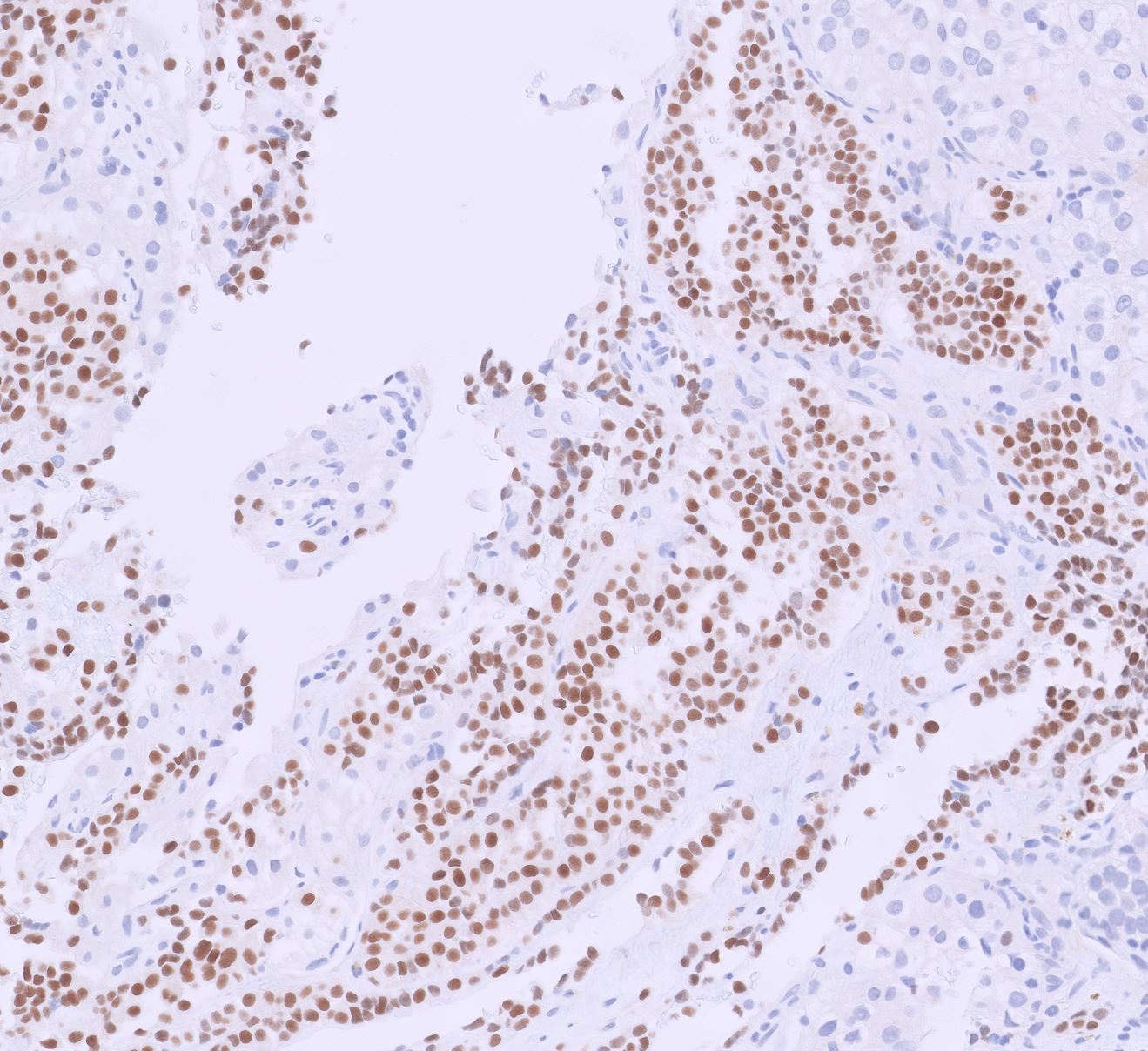


***SF-1, 20X***

- **Case Report 2**

***Diagnosis:***

Sellar tumor, excision:

- Metastatic adenocarcinoma.

- Pituitary adenoma.

***Comments:***

Sections show sheets of cells with diffuse effacement of the normal lobular architecture, as highlighted by special stain for reticulin. Also present is a somewhat well-circumscribed lesion with prominent nucleoli forming glands and cribriform spaces with necrosis.

Immunohistochemical stains show the sheets of neoplastic cells to be positive for chromogranin, scattered weak positivity for p53, scattered positive FSH and alpha-subunit of HCG, and negative for ACTH, TSH, Prolactin, and hGH. The Ki67 shows a proliferation rate of approximately 1%. These findings are consistent with the diagnosis of a pituitary adenoma.

The glandular neoplastic cells are positive for CAM 5.2, PSA-431, NKX3.1, and Androgen receptor, and negative for CK7, CK20, CDX2, TTF1, Napsin A, Hepatocyte, Villin, RCC, and PAX8. These findings are most consistent with a metastatic adenocarcinoma from a prostate primary.


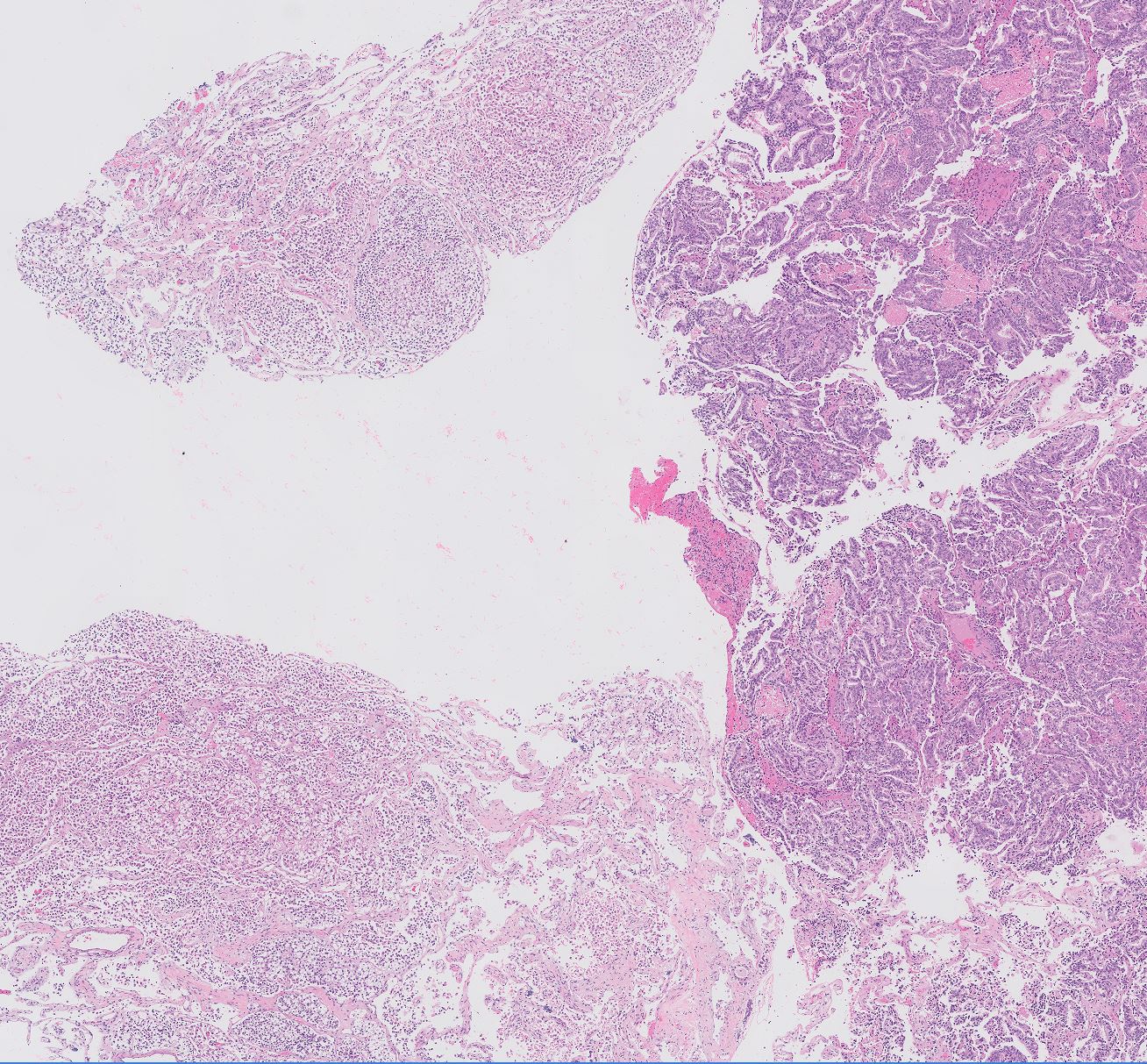


***Hematoxylin and Eosin, 2X***


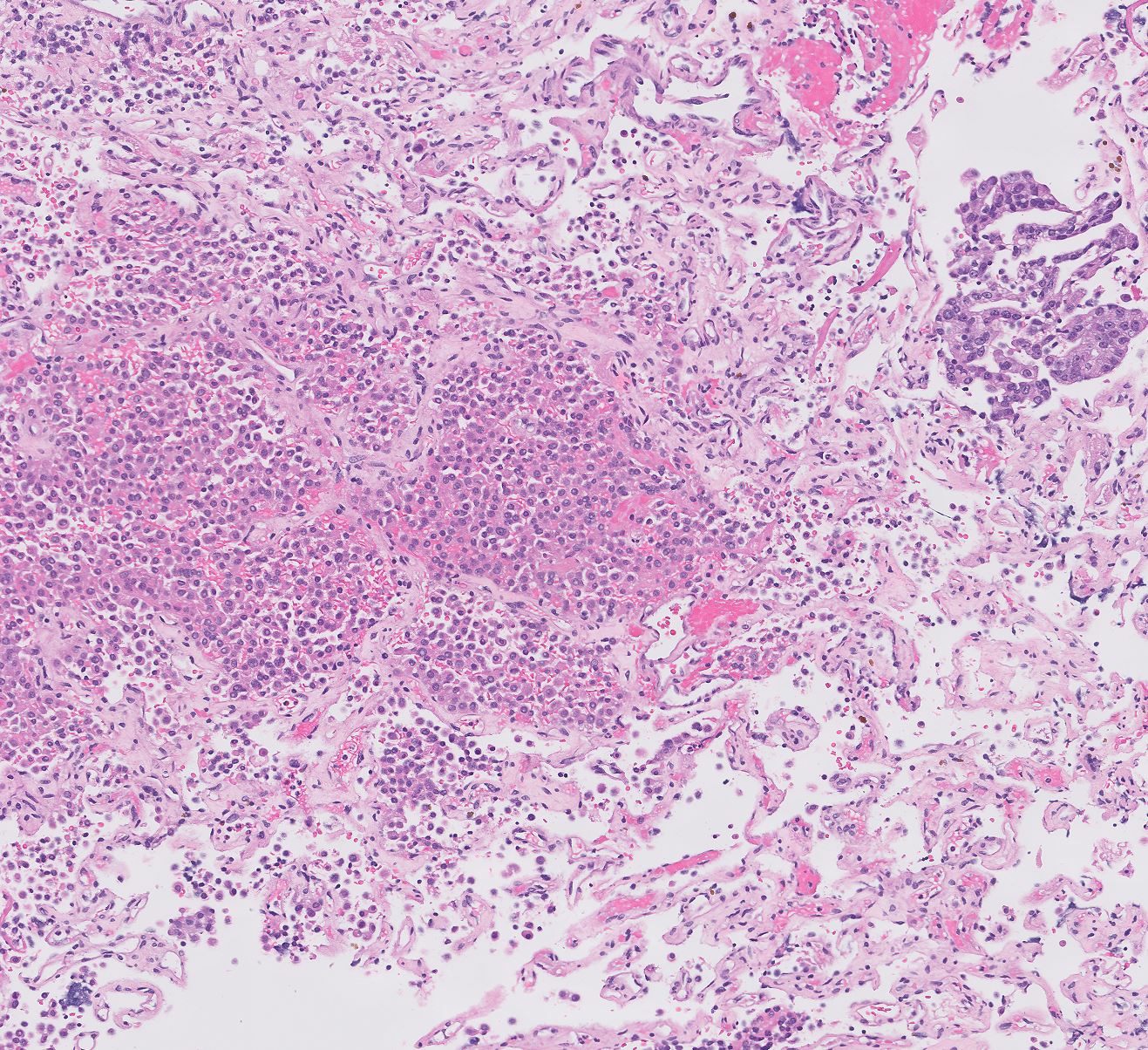


***Hematoxylin and Eosin, 10X***


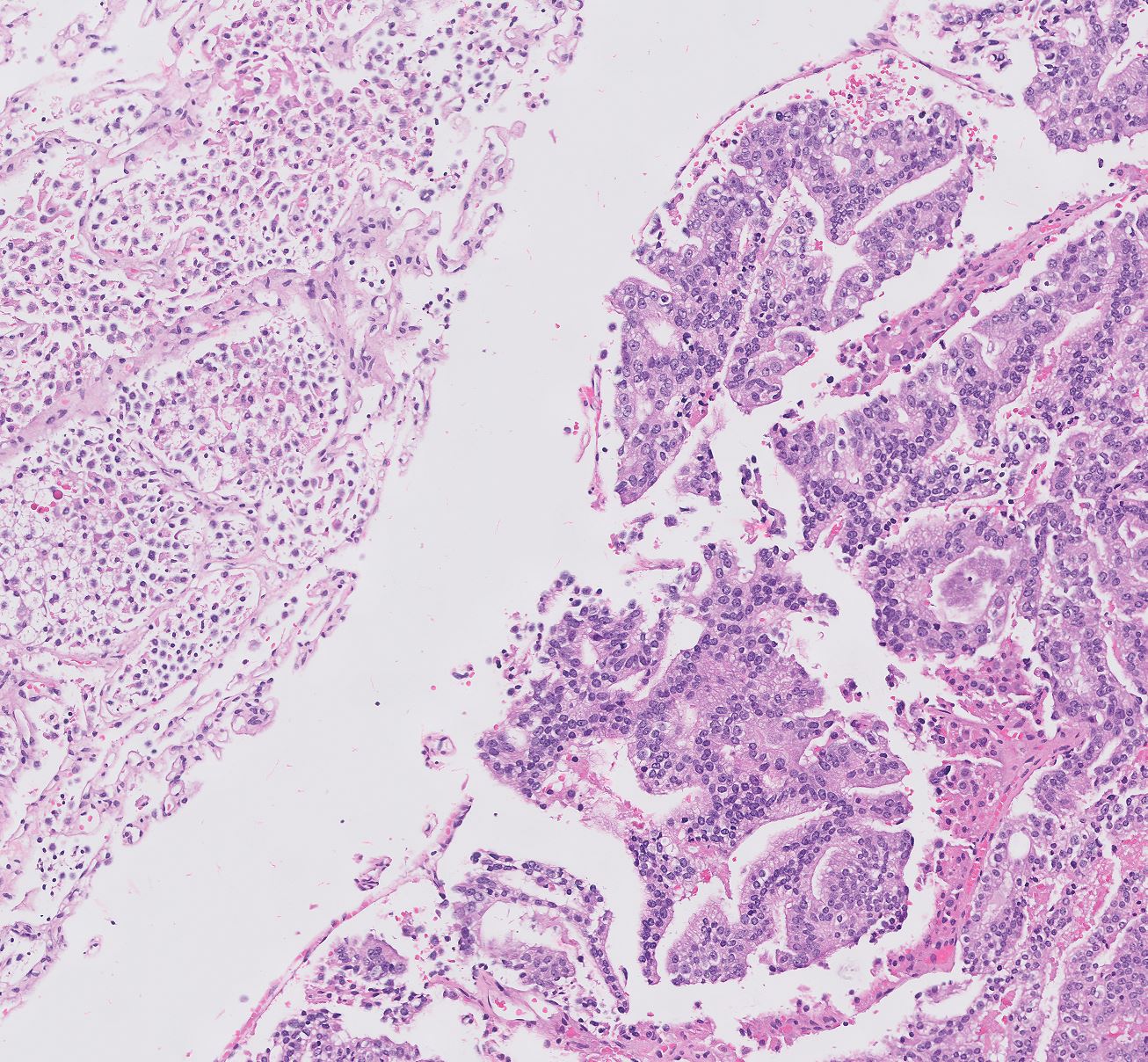


***Hematoxylin and Eosin, 10X***


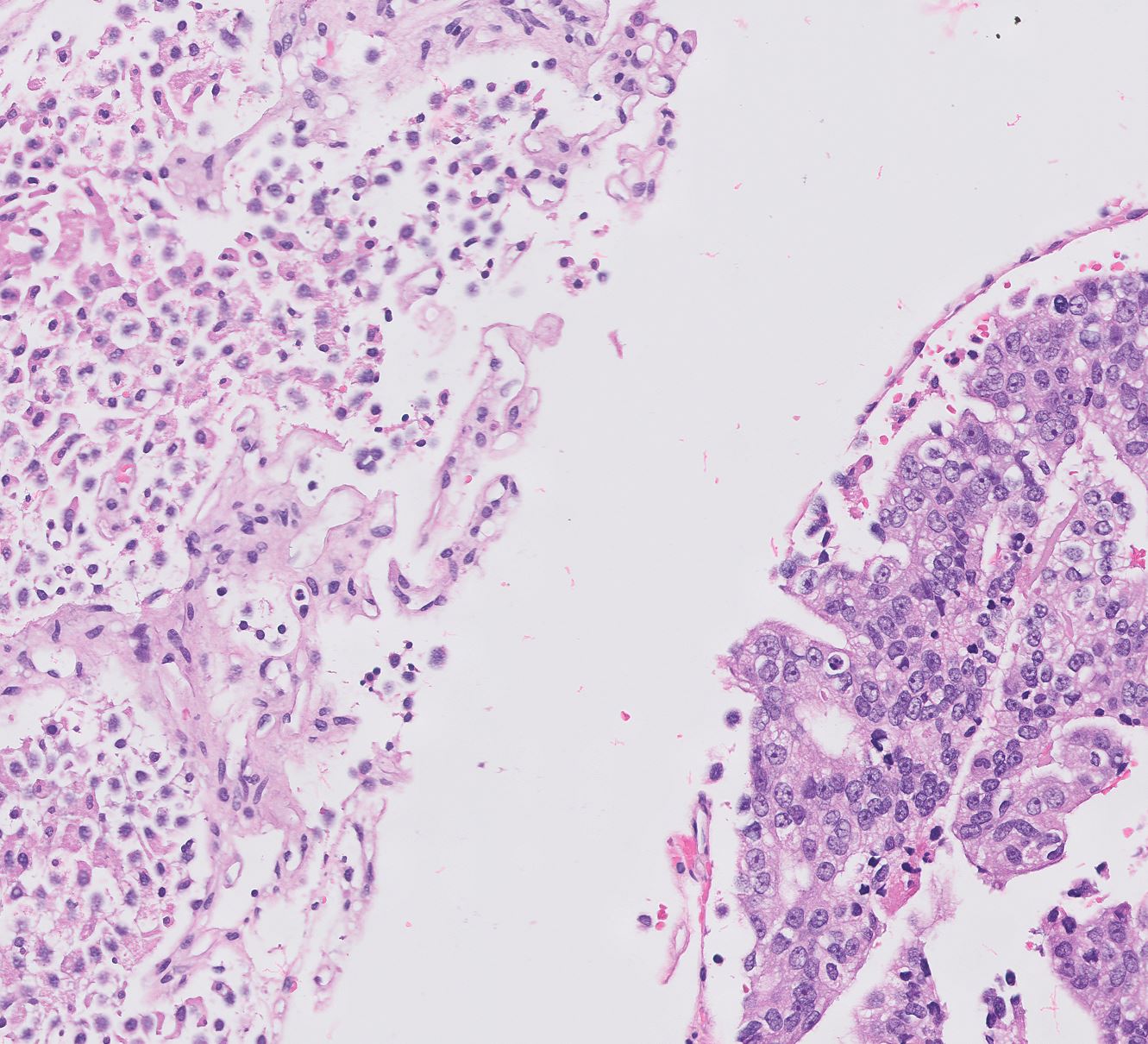


***Hematoxylin and Eosin, 20X***


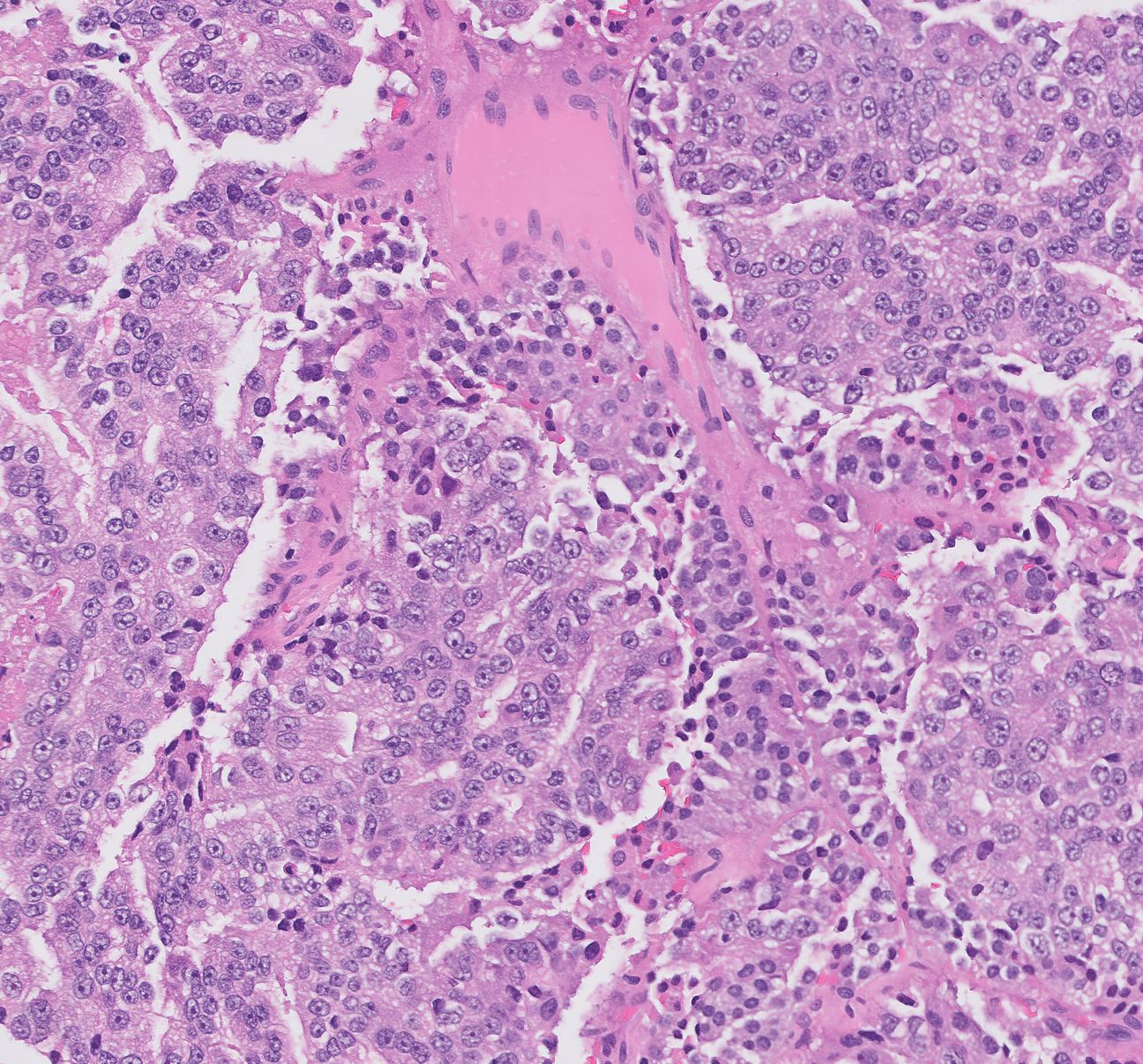


***Hematoxylin and Eosin, 20X***


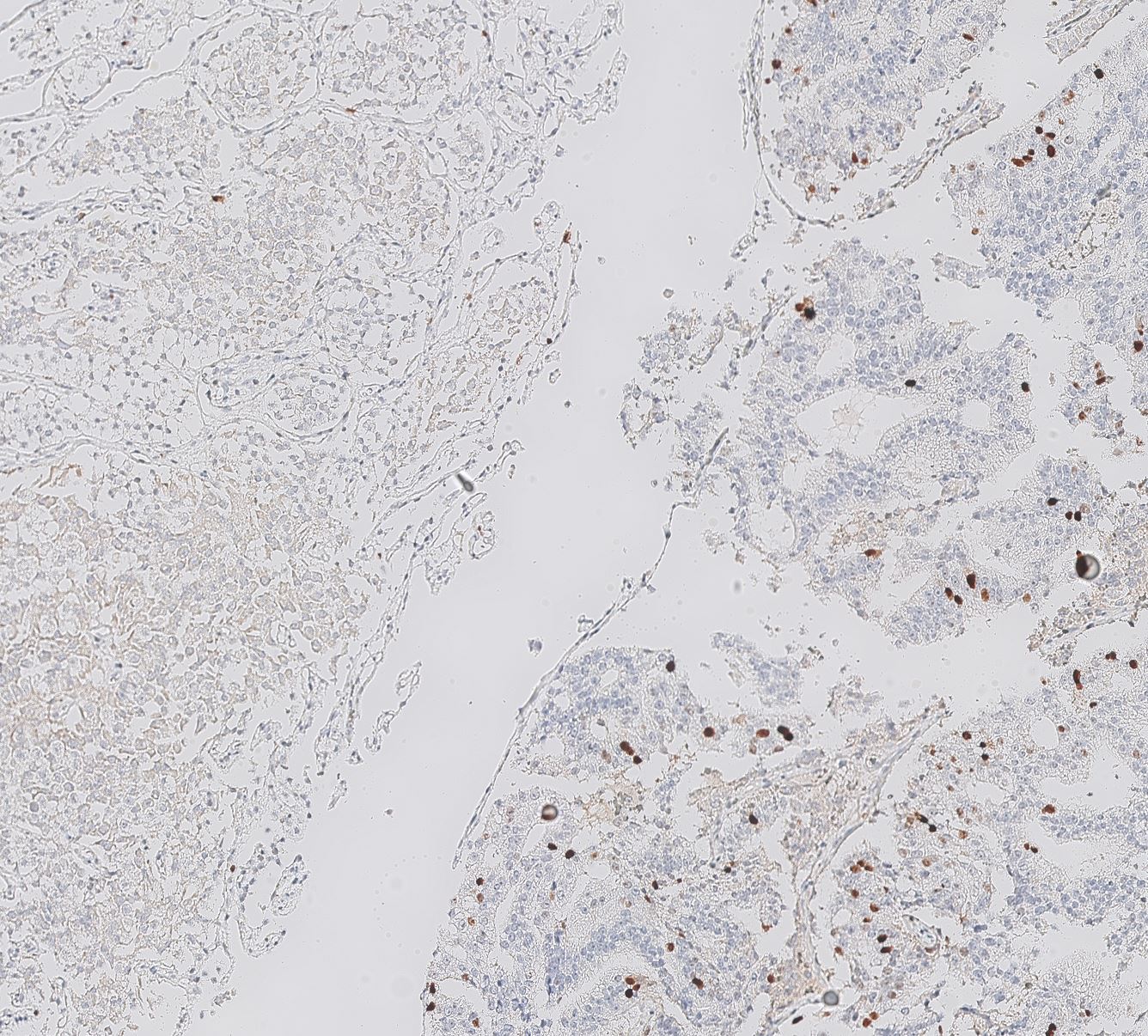


***Ki-67, 10X***


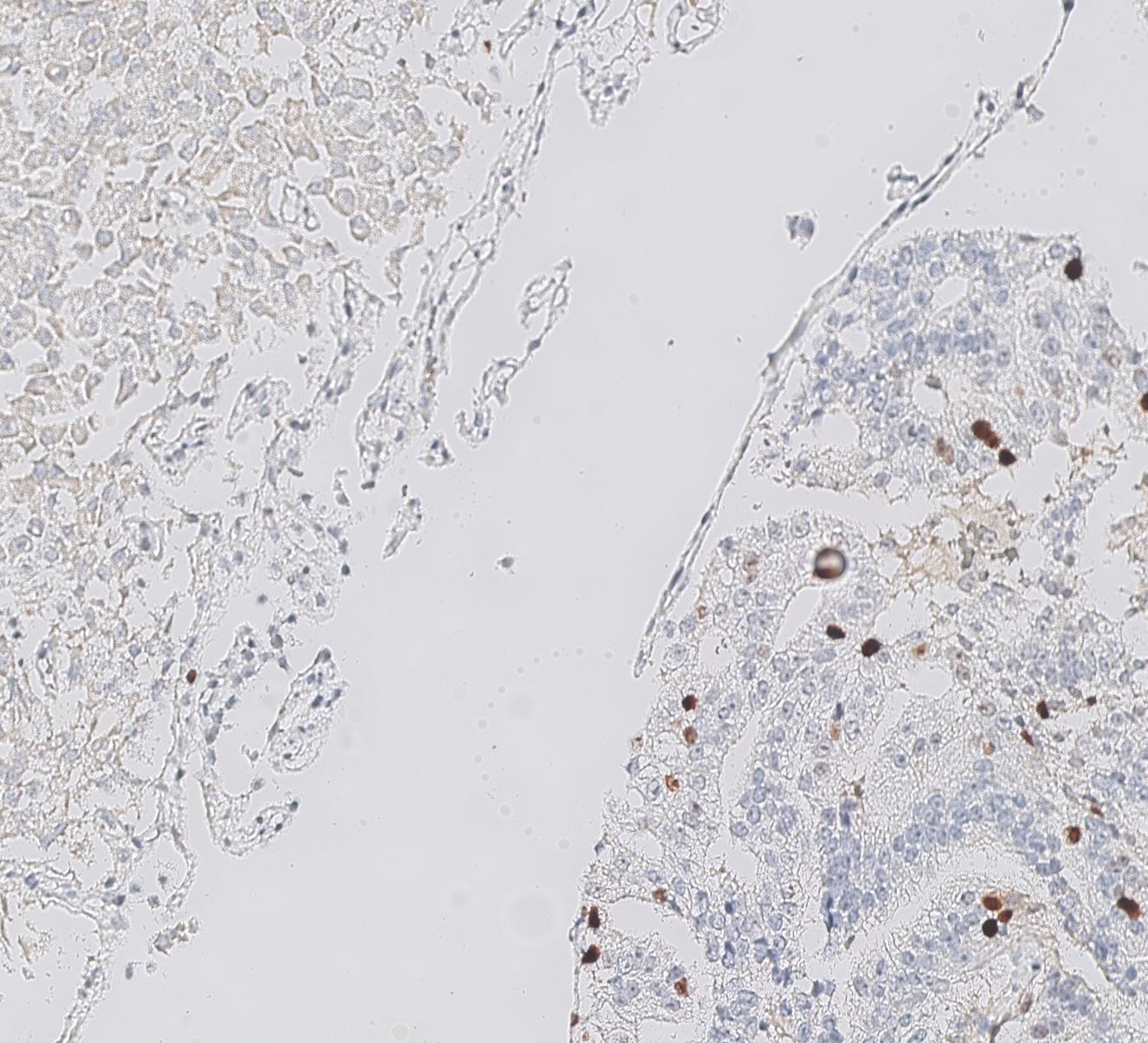


***Ki-67, 20X***


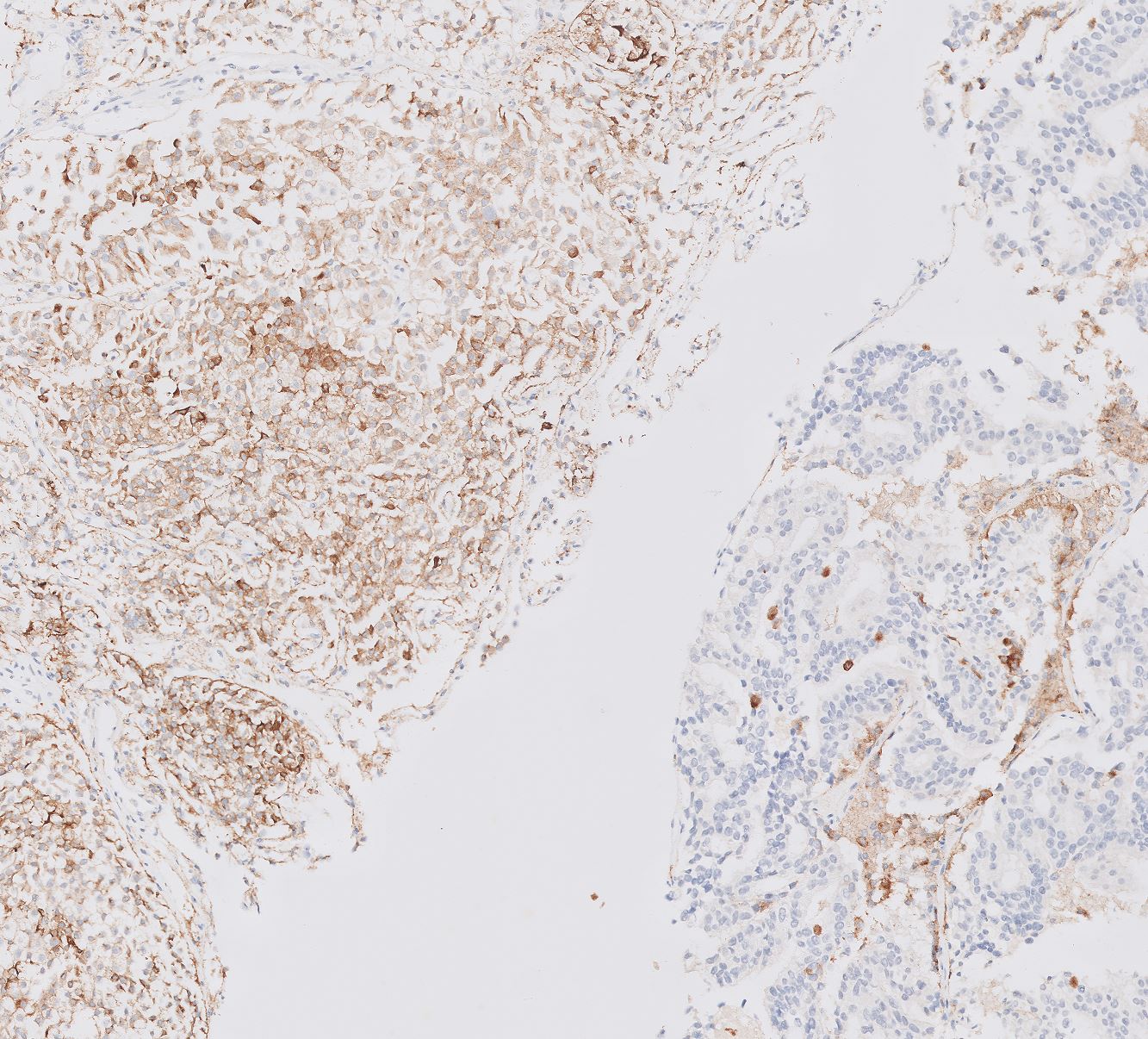


***Chromogranin, 10X***


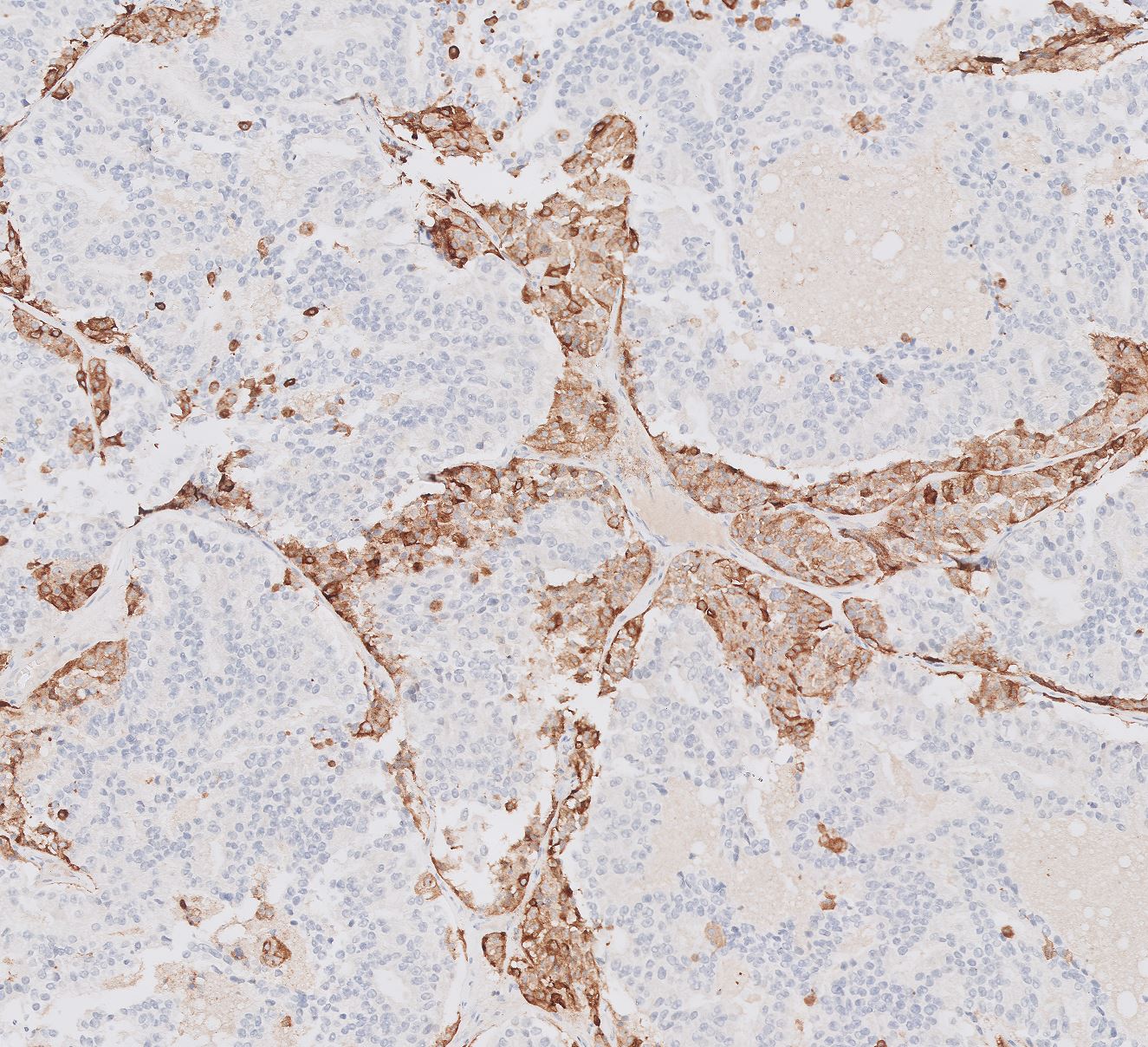


***Chromogranin, 10X***


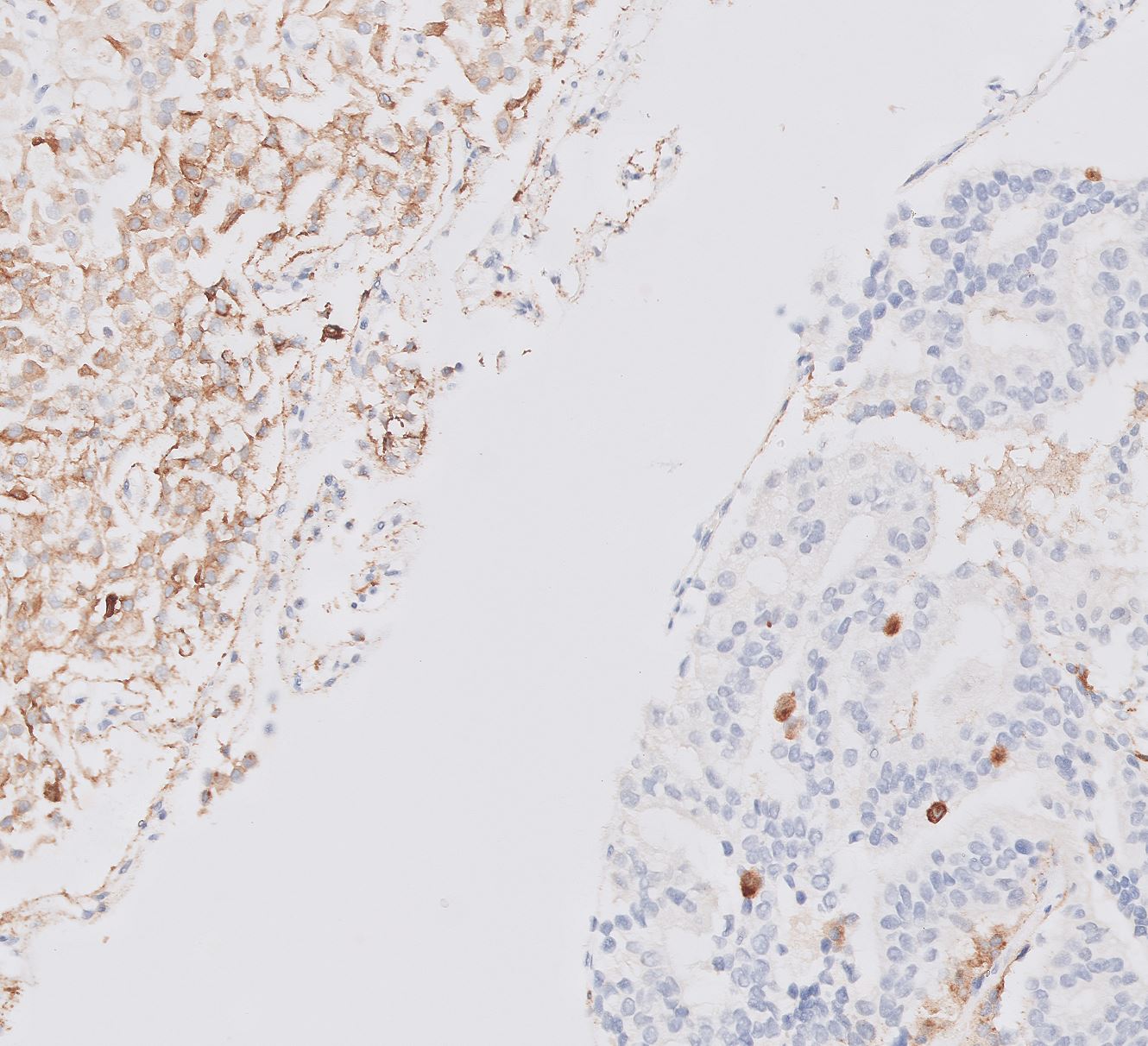


***Chromogranin, 20X***


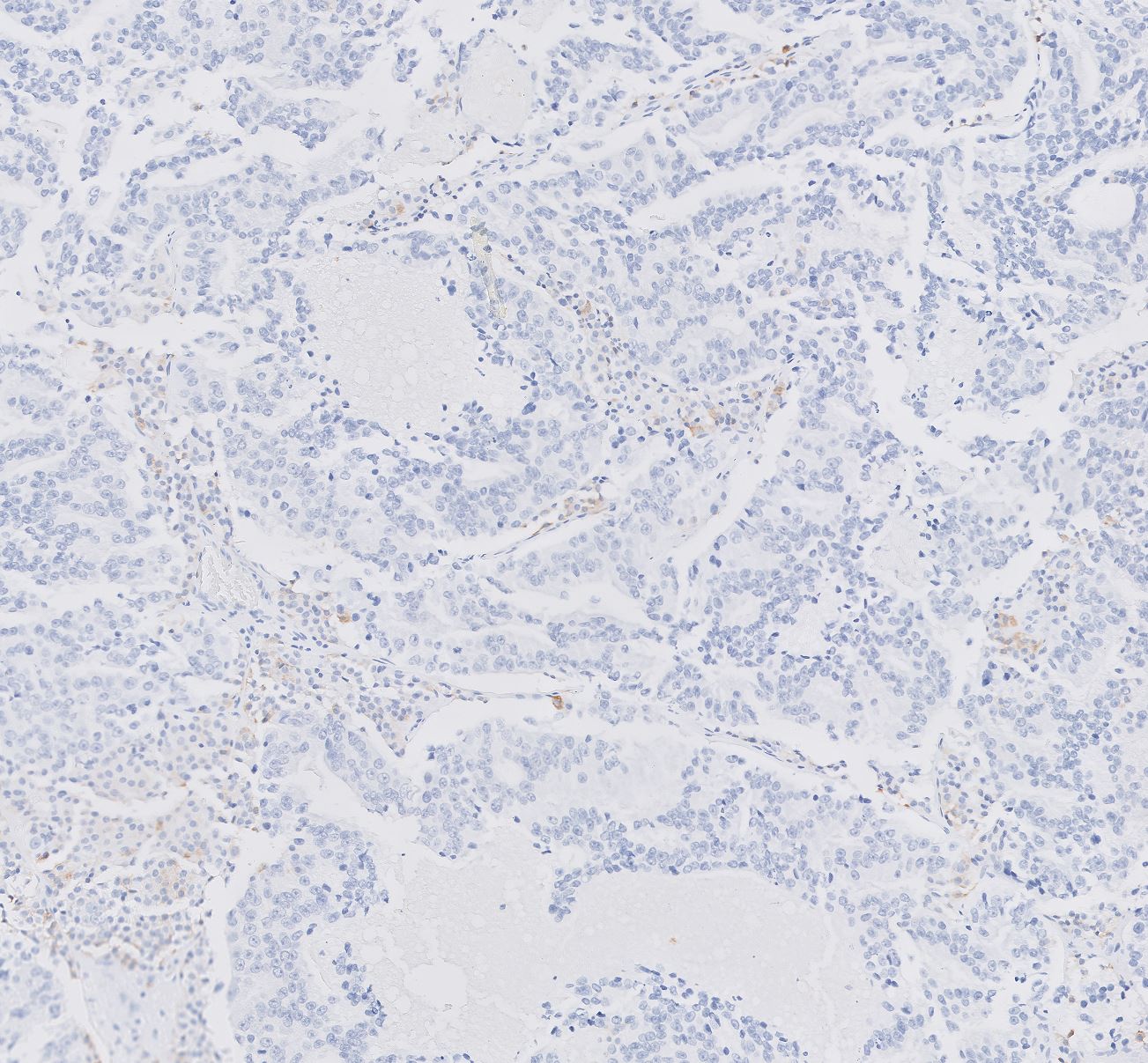


***FSH, 10X***


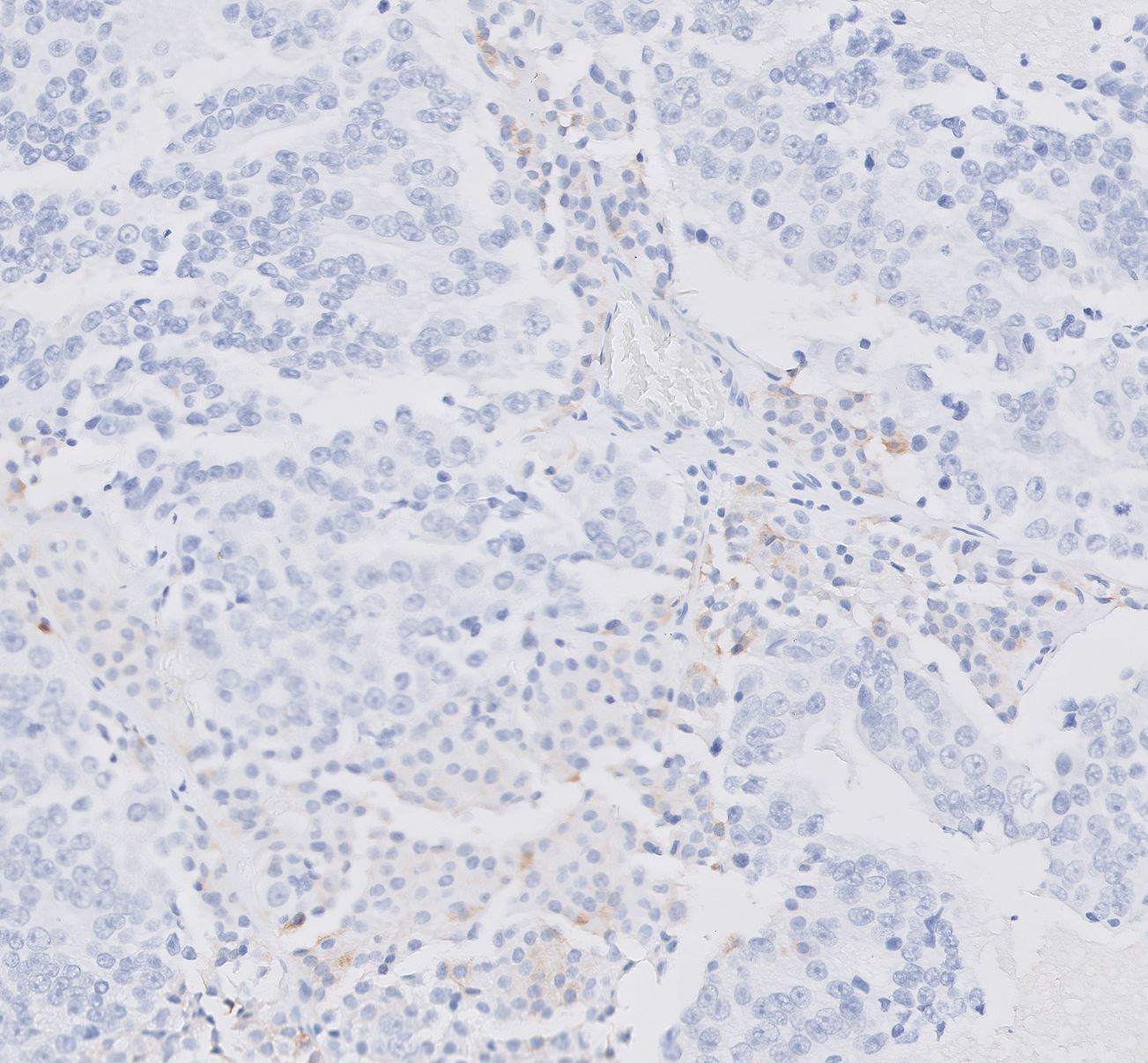


***FSH, 20X***

***
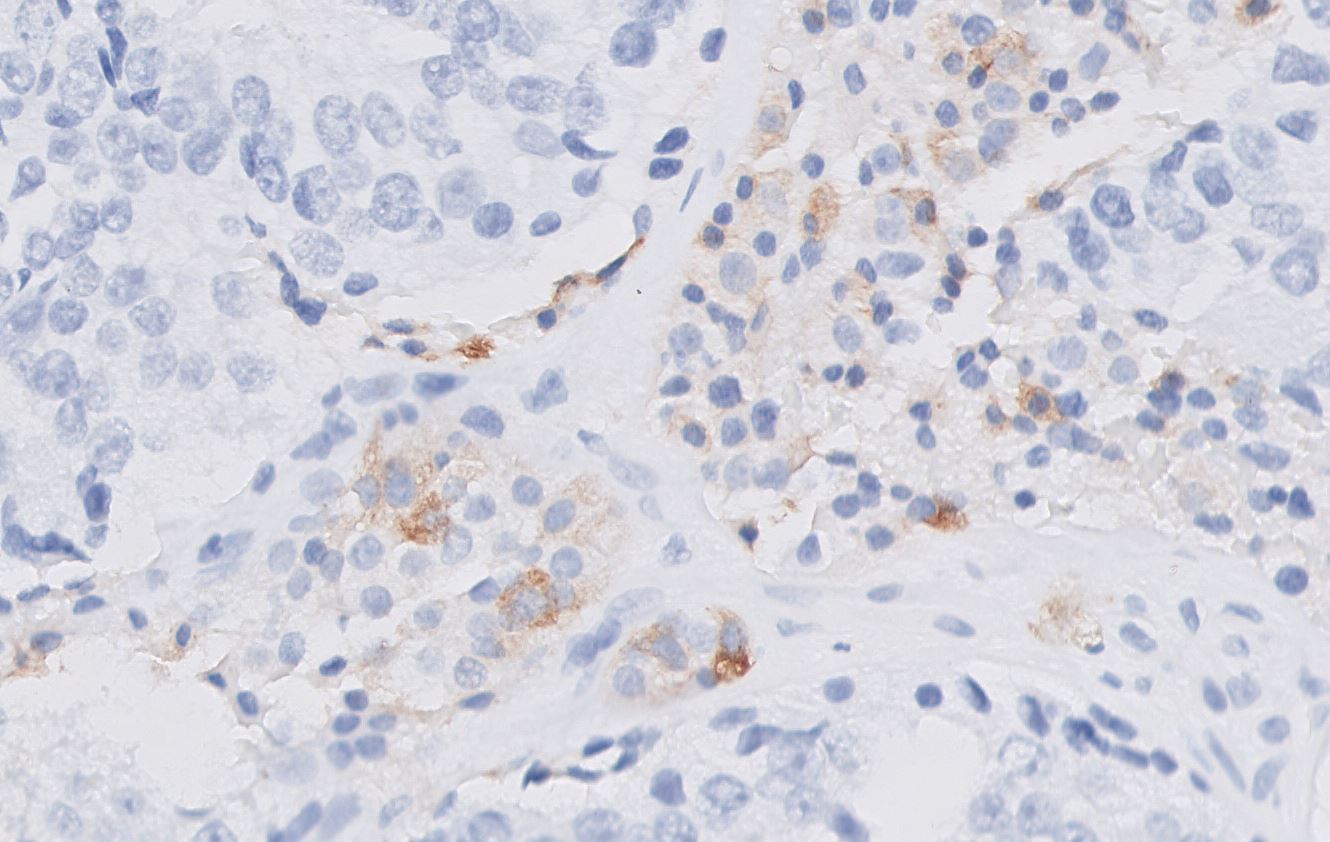
***

***FSH, 40X***

***
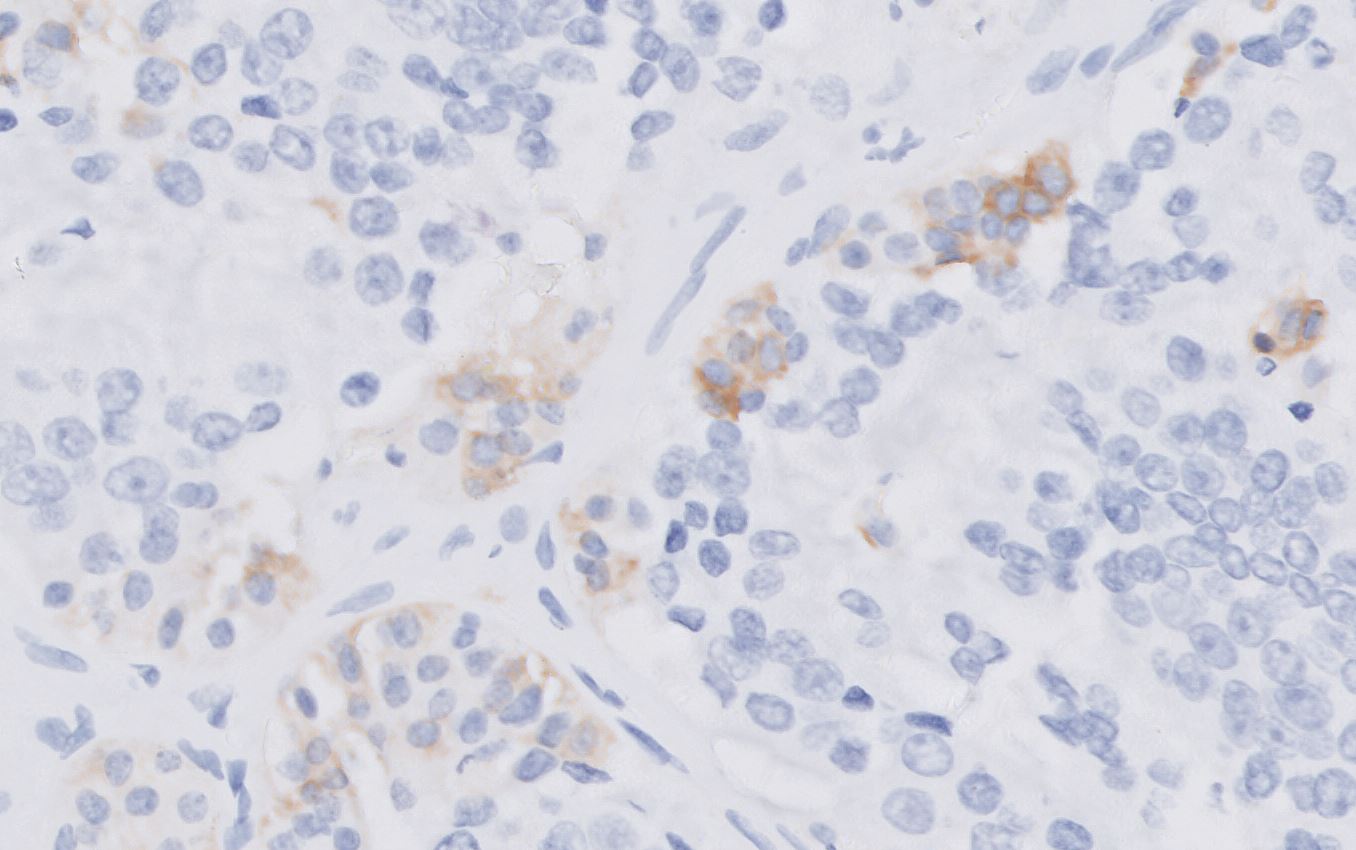
***

***FSH, 40X***

***
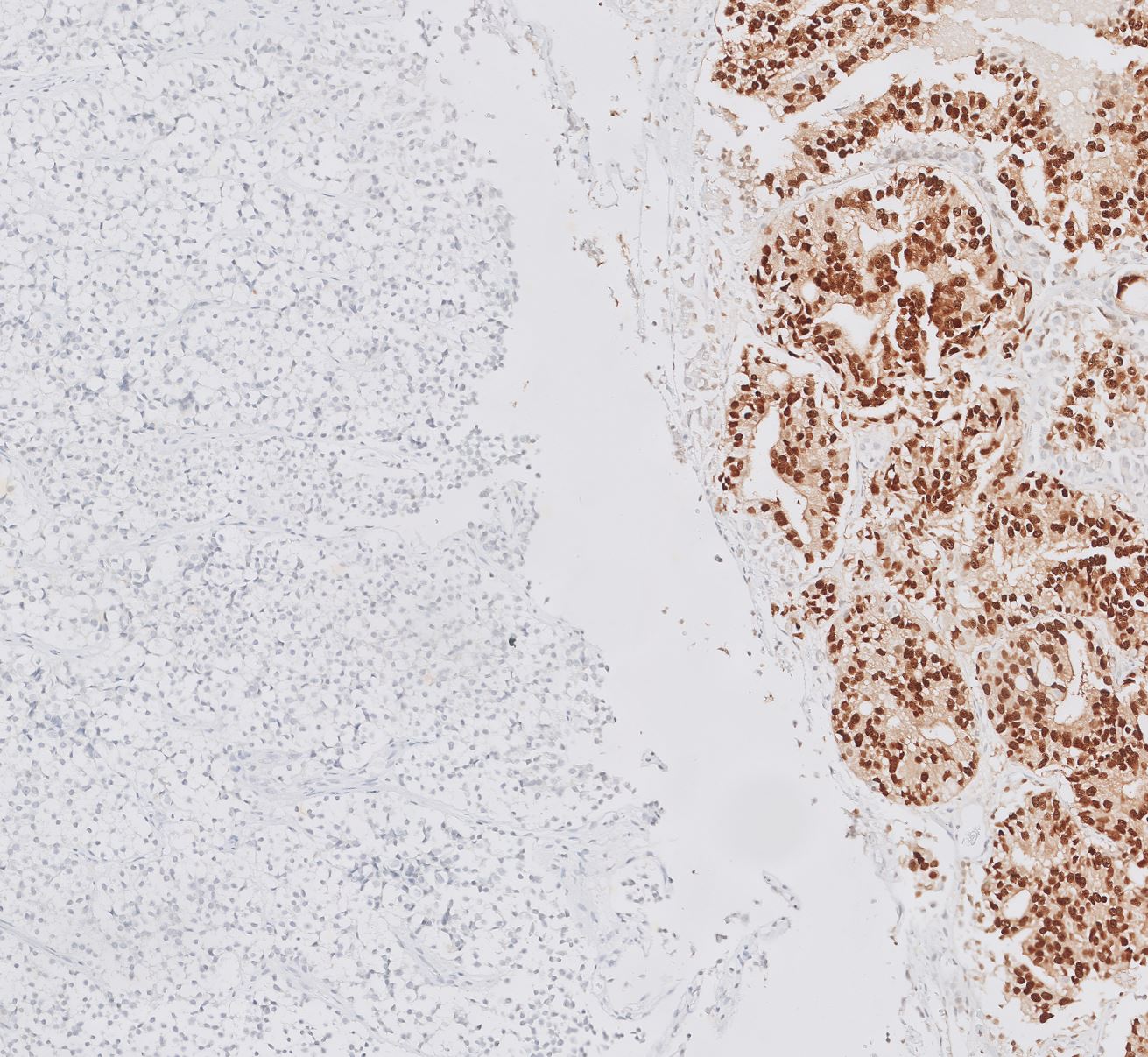
***

***NKX3.1, 10X***

***
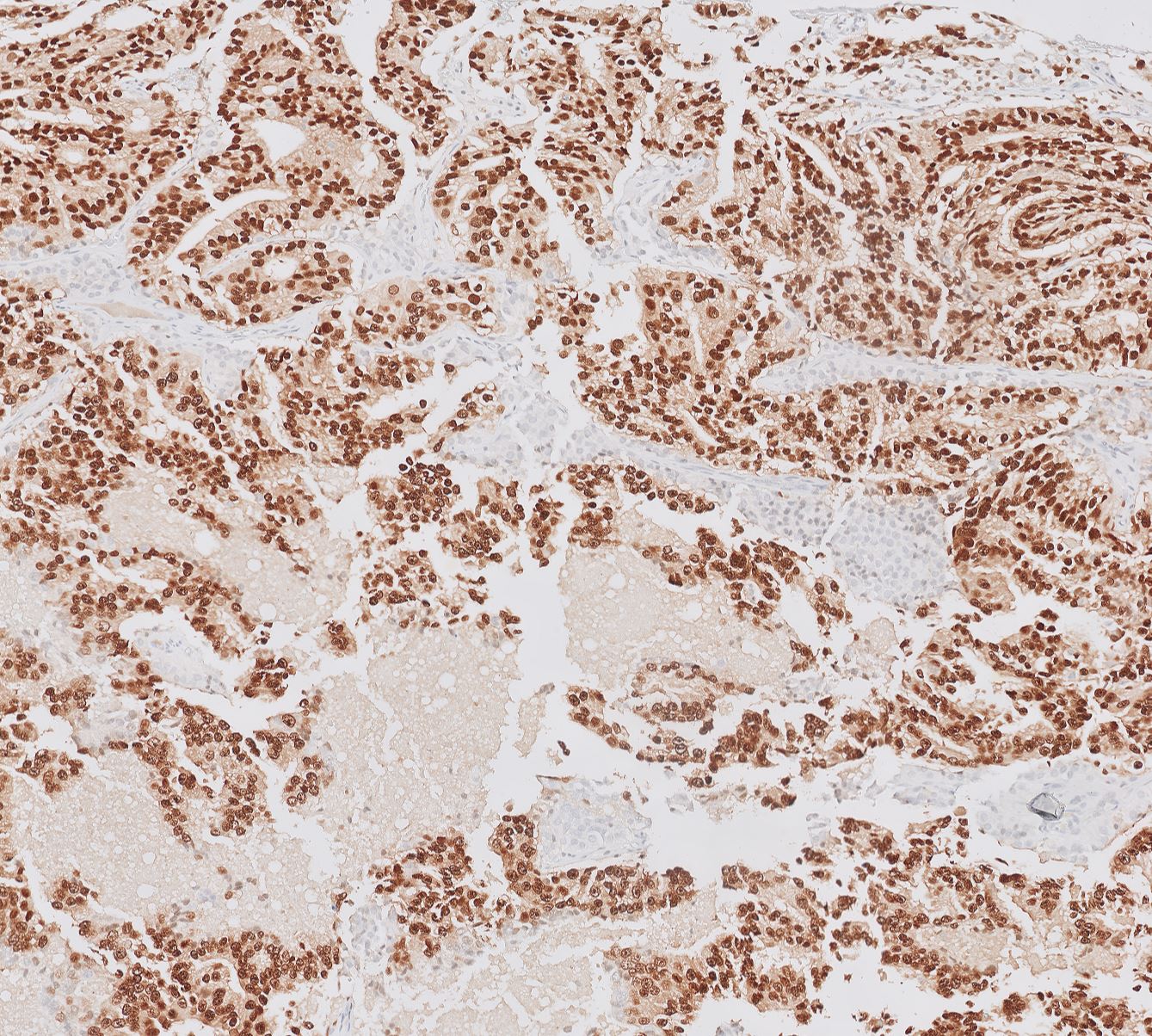
***

***NKX3.1, 10X***

***
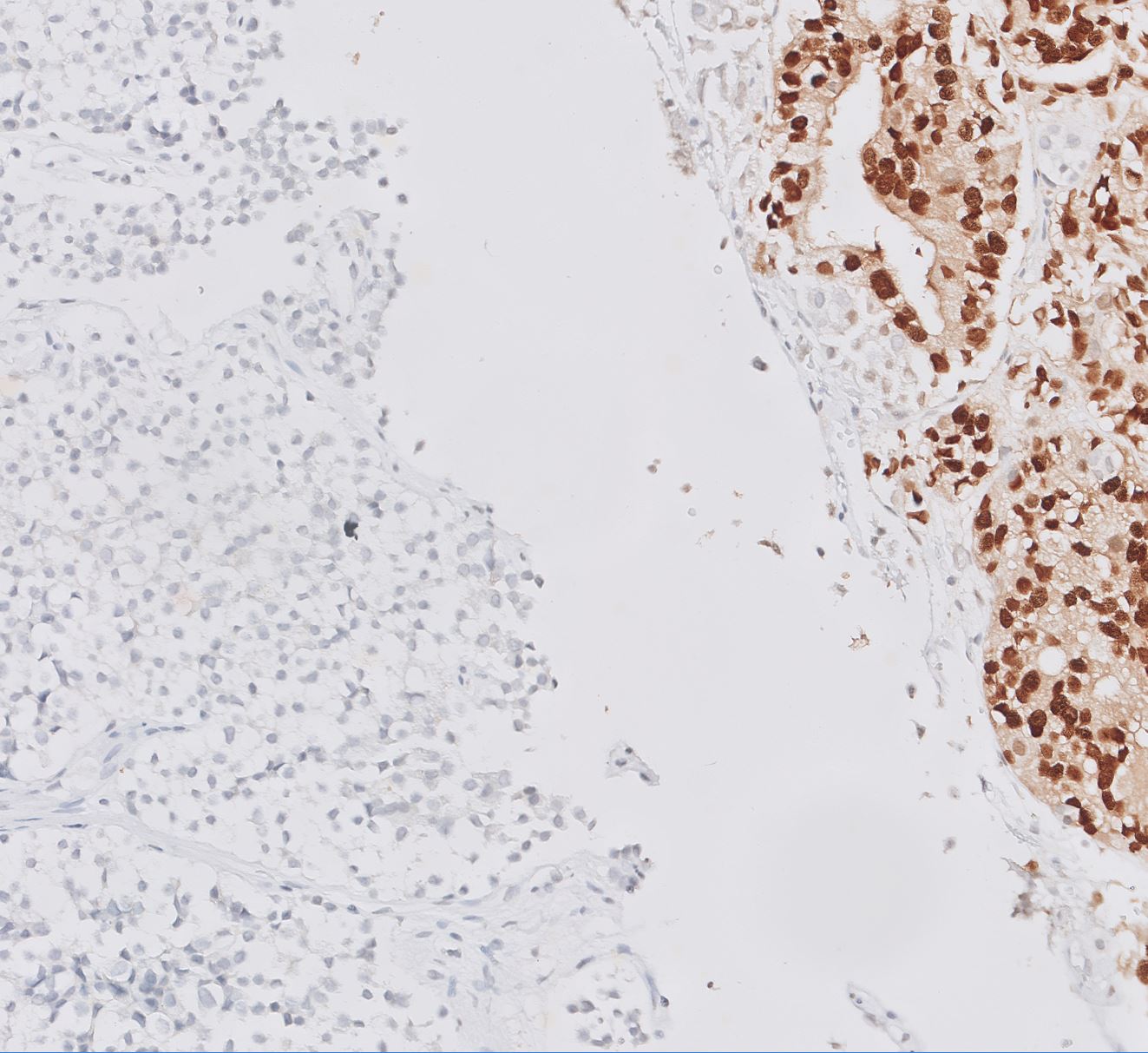
***

***NKX3.1, 20X***

***
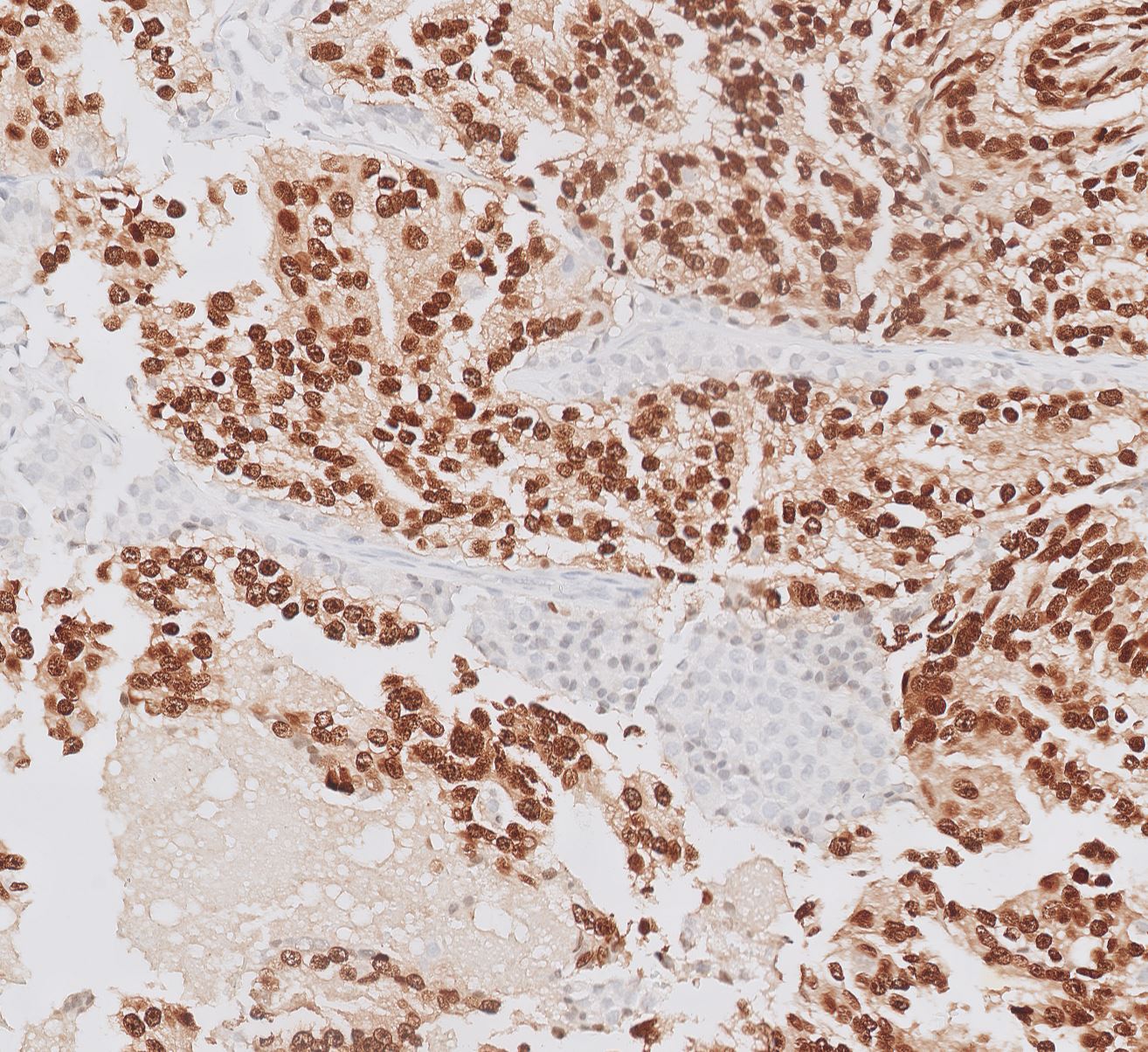
***

***NKX3.1, 20X***
