# Supplementary material for: “Intrasellar tumor-to-tumor metastasis: A single center experience with a systematic review”
Source: Pituitary. 2024 Aug 14;27(5):455–67. doi: 10.1007/s11102-024-01441-9 (PMC11513765; doi:10.1007/s11102-024-01441-9)
Supplement: Supplementary file 2 — Supplementary file2 (DOCX 31 KB) [file 11102_2024_1441_MOESM2_ESM.docx]

| **Study, first author** | **Primary site** | **Age** | **Gender** | **Type of adenoma** | **Size (mm) - Longest axis** | **Visual deficit** | **Headache** | **Diplopia** | **Hypopituitarism** | **Acromegaly** | **Fever** | **Hemiparesis** | **Hypercortisolism** | **Outcome** | **Follow-up time (months)** | **Alive** | **Type of surgery** | **Radiation** |
| --- | --- | --- | --- | --- | --- | --- | --- | --- | --- | --- | --- | --- | --- | --- | --- | --- | --- | --- |
| Andreev et al, 2020 [35] | Breast | 55 | F | FSH/ LH | NA | Yes | No | Yes | No | No | No | No | No | Subtotal | NA | Y | TSS | N |
| Bret et al, 2001 [37] | Breast | 75 | F | FSH/ LH | NA | Yes | No | No | No | No | No | No | No | NA | 18 | NA | EEA | NA |
| Richardson and Katayama, 1971 [38] | Breast | 70 | F | Histology not provided | NA | No | Yes | No | No | No | No | Yes | No | NA | 6 | Y | EEA | N |
| Van der Zwan et al, 1971 [39] | Breast | 73 | F | Histology not provided | NA | Yes | No | No | No | No | No | No | No | NA | 0.4 | Y | EEA | N |
| Zager et al, 1987 [41] | Breast | 56 | F | FSH/ LH | NA | No | No | Yes | No | No | No | No | No | Autopsy | 0.933 | N | NA | NA |
| Mills et al, 2018 [40] | Breast | 65 | F | FSH | 27 | Yes | Yes | No | No | No | No | No | No | NA | 3 | Y | EEA | Y |
| Castle-Kirszbaum et al, 2020 [36] | Breast | 51 | F | Null cell | NA | Yes | No | No | Yes | No | No | No | No | Subtotal | NA | N | EEA | Y |
| Noga et al, 2001 [59] | Colon | 60 | M | Null cell | 65 | Yes | No | No | No | No | No | No | No | Subtotal | 9 | N | TSS | Y |
| Skulsampaopol et al, 2017 [60] | Colon | 48 | M | Histology not provided | 22 | Yes | No | No | Yes | No | No | No | No | Subtotal | Few days | Y | EEA | Y |
| Thewjitcharoen, 2014 [57] | Colon | 65 | M | Prolactinoma | 23 | Yes | Yes | Yes | Yes | No | No | No | No | Subtotal | 3 | NA | TSS | NA |
| Donofrio et al, 2020 [58] | Colon | 68 | M | FSH/ LH | NA | Yes | No | No | No | No | No | No | No | GTR | 9 | N | TSS | N |
| Gariépy et al, 2023 [65] | Esophagus | 65 | M | Prolactinoma | 31 | No | No | Yes | No | No | No | No | No | Subtotal | 4 | N | TSS | N |
| Mollinati et al, 1985 [49] | Gastric | 66 | F | GH | NA | Yes | No | No | No | No | No | No | No | NA | 0.067 | NA | TSS | Y |
| van Seters et al, 1985 [61] | Gastric | 66 | F | Prolactinoma | NA | Yes | No | No | Yes | No | No | No | No | Subtotal | 0.4 | N | TSS | NA |
| Sogani et al, 2014 [42] | Lung | 64 | M | ACTH | 16 | Yes | No | No | Yes | No | No | No | No | Subtotal | 7 | N | TSS | N |
| Rotondo et al, 2013 [46] | Lung | 66 | M | Prolactinoma | 19 | No | Yes | No | No | No | No | No | No | Autopsy | NA | N | EEA | N |
| Hoellig et al, 2009 [47] | Lung | 71 | M | Null cell | 34 | Yes | No | No | No | No | No | No | No | Subtotal | 0.433 | N | TC | N |
| Mollinati et al, 1985 [49] | Lung | 71 | M | Histology not provided | NA | Yes | No | No | No | No | No | No | No | NA | 1 | N | TSS | Y |
| Post et al, 1988 [50] | Lung | 77 | M | Histology not provided | NA | Yes | No | Yes | Yes | No | No | No | No | Subtotal | NA | NA | TSS | Y |
| Hanna et al, 1999 [48] | Lung | 42 | F | Prolactinoma | NA | No | Yes | No | No | No | No | No | No | NA | 6 | N | TSS | Y |
| Nasr et al, 2006 [52] | Lung | 44 | F | GH | NA | Yes | No | No | No | Yes | No | No | No | NA | NA | N | TC | N |
| Fujimori et al, 2014 [51] | Lung | 80 | M | Histology not provided | NA | Yes | No | Yes | No | No | No | No | No | NA | 2 | Y | EEA | Y |
| Suzuki et al, 2023 [44] | Lung | 75 | M | FSH/ LH | 32 | Yes | No | Yes | Yes | No | No | No | No | Subtotal | 12 | N | EEA | Y |
| Abe at al, 1997 [62] | Mediastinum | 46 | F | Prolactinoma | 45 | Yes | Yes | No | No | No | No | No | No | NA | 6 | N | NA | Y |
| Yang et al, 2017 [63] | Melanoma | 62 | F | Prolactinoma | 30 | Yes | Yes | No | No | No | No | No | No | NA | 1 | NA | TSS | N |
| Jung et al, 2007 [64] | Melanoma | 75 | M | Null cell | 62 | Yes | No | No | Yes | No | No | No | No | NA | 22 | N | TSS | Y |
| Ramsay et al, 1988 [66] | Pancreas | 50 | F | ACTH | NA | No | No | No | No | No | No | No | Yes | NA | 8 | N | NA | N |
| Ramsay et al, 1988 [66] | Prostate | 57 | M | Null cell | NA | No | No | No | No | No | No | No | No | Autopsy | 12 | N | TC | Y |
| James et al, 1984 [53] | Renal | 75 | M | Null cell | 20 | Yes | No | No | No | No | No | No | No | NA | 120 | N | NA | N |
| Weber et al, 2003 [54] | Renal | 62 | F | Null cell | NA | Yes | No | No | No | No | No | No | No | Subtotal | 1 | N | EEA | Y |
| Burns et al, 1973 [55] | Renal | 78 | M | Histology not provided | NA | No | No | No | No | No | Yes | No | No | NA | NA | Y | EEA | Y |
| Magnoli et al, 2014 [56] | Renal | 75 | F | FSH/ LH | NA | Yes | No | Yes | No | No | No | No | No | GTR | 24 | Y | EEA | Y |
| Post et al, 1988 [50] | Unidentified | 61 | F | ACTH | NA | Yes | Yes | No | Yes | No | No | No | No | Subtotal | 1 | N | EEA | N |
| Bret et al, 2001 [37] | Unidentified | 87 | F | FSH/ LH | NA | Yes | No | No | No | No | No | No | No | NA | 8 | N | TC | Y |
| Nassiri et al, 2012 [68] | Unidentified | 55 | F | GH | 30 | No | No | No | No | Yes | No | No | No | Subtotal | 6 | N | TC | N |
| Hurley et al, 1992 [67] | Unidentified | 76 | M | GH | NA | Yes | No | No | No | Yes | No | No | No | NA | 6 | N | TC | NA |
| Our case 1 | Renal | 56 | M | FSH/ LH | 64 | Yes | No | No | Yes | No | No | No | No | Subtotal | 5 | Y | EEA | N |
| Our case 2 | Prostate | 72 | M | FSH | 26 | No | No | No | No | No | No | No | No | GTR | NA | N | NA | Y |

**Supplementary Table 1:** Summary of case reports with malignant tumors metastasis within PitNETs.

M, Male; F, Female; Y, Yes; N. No; N/A, Not Available; GTR, Gross Total Resection; EEA, Endoscopic Endonasal Approach; TSS, Transsphenoidal; TC, Transcranial; GH, Growth Hormone; ACTH, Adrenocorticotrophic Hormone.
