# Supplementary material for: “Intrasellar tumor-to-tumor metastasis: A single center experience with a systematic review”
Source: Pituitary. 2024 Aug 14;27(5):455–67. doi: 10.1007/s11102-024-01441-9 (PMC11513765; doi:10.1007/s11102-024-01441-9)
Supplement: Supplementary file 3 — Supplementary file3 (DOCX 28 KB) [file 11102_2024_1441_MOESM3_ESM.docx]

**Supplementary** **Table 2:** Risk of bias assessment among included studies

| Study, first author | Year | Pt no. | Demographics Described (0=no, 1=yes, 2=unclear) | Patient history presented as timeline (0=no, 1=yes, 2=unclear) | Description of current clinical condition (0=no, 1=yes, 2=unclear) | Description of diagnostic tests and results (0=no, 1=yes, 2=unclear) | Description of intervention/treatment (0=no, 1=yes, 2=unclear) | Descripton of post-intervention condition (0=no, 1=yes, 2=unclear) | Description of adverse events (0=no, 1=yes, 2=unclear) | Takeaway lessons (0=no, 1=yes, 2=unclear) | Risk of Bias |
| --- | --- | --- | --- | --- | --- | --- | --- | --- | --- | --- | --- |
| *Abe et al [62]* | 1997 | 1 | Y | Y | Y | Y | Y | Y | Y | Y | Low |
| *Andreev et al [35]* | 2020 | 1 | Y | Y | N | Y | Y | Y | Y | Y | Low |
| *Bret et al [37]* | 2001 | 2 | Y | Y | Y | Y | Y | Y | Y | Y | Low |
| *Burns et al [55]* | 1973 | 1 | Y | Y | N | Y | Y | Y | N | Y | Low |
| *Castle-Kirszbaum et al [36]* | 2020 | 1 | Y | N | N | Y | Y | Y | Y | Y | Low |
| *Donofrio et al [58]* | 2020 | 1 | Y | Y | Y | Y | Y | Y | Y | Y | Low |
| *Fujimori et al [51]* | 2014 | 1 | Y | Y | Y | N | Y | N | Y | Y | Low |
| *Gariépy et al [65]* | 2023 | 1 | Y | Y | N | Y | Y | Y | N | Y | Low |
| *Hanna et al [48]* | 1999 | 1 | Y | Y | N | Y | Y | N | Y | Y | Low |
| *Hoellig et al [47]* | 2009 | 1 | Y | Y | Y | Y | Y | Y | Y | Y | Low |
| *Hurley et al [67]* | 1992 | 1 | Y | Y | Y | Y | Y | Y | Y | Y | Low |
| *James et al [53]* | 1984 | 1 | Y | Y | N | Y | Y | N | N | Y | Medium |
| *Jung et al [64]* | 2007 | 1 | Y | Y | N | Y | Y | Y | Y | Y | Low |
| *Magnoli et al [56]* | 2014 | 1 | Y | Y | Y | Y | Y | Y | Y | Y | Low |
| *Mills et al [40]* | 2018 | 1 | Y | N | Y | Y | Y | Y | Y | Y | Low |
| *Mollinati et al [49]* | 1985 | 2 | Y | Y | Y | Y | Y | Y | Y | Y | Low |
| *Nasr et al [52]* | 2006 | 1 | Y | Y | Y | Y | Y | Y | Y | Y | Low |
| *Nassiri et al [68]* | 2012 | 1 | Y | Y | Y | Y | Y | Y | Y | Y | Low |
| *Noga et al [59]* | 2001 | 1 | Y | Y | Y | Y | Y | Y | Y | Y | Low |
| *Post et al [50]* | 1988 | 2 | Y | N | Y | Y | Y | N | N | Y | Medium |
| *Ramsay et al [66]* | 1988 | 2 | Y | Y | Y | Y | N | N | Y | Y | Low |
| *Richardson and Katayama [38]* | 1971 | 1 | Y | Y | Y | Y | Y | Y | Y | Y | Low |
| *Rotondo et al [46]* | 2013 | 1 | Y | Y | Y | Y | Y | N | Y | Y | Low |
| *Skulsampaopol et al [60]* | 2017 | 1 | Y | N | Y | Y | Y | Y | Y | Y | Low |
| *Sogani et al [46]* | 2014 | 1 | Y | N | N | Y | Y | N | N | Y | Medium |
| *Suzuki et al [44]* | 2024 | 1 | Y | Y | Y | Y | Y | Y | Y | Y | Low |
| *Thewjitcharoen et al [57]* | 2014 | 1 | Y | N | Y | Y | Y | N | Y | Y | Low |
| *Van der Zwan et al [39]* | 1971 | 1 | Y | N | Y | Y | N | Y | Y | Y | Low |
| *Van Seters et al [61]* | 1985 | 1 | Y | Y | Y | Y | Y | Y | Y | Y | Low |
| *Weber et al [54]* | 2003 | 1 | Y | N | Y | Y | Y | Y | Y | Y | Low |
| *Yang et al [63]* | 2017 | 1 | Y | Y | Y | Y | Y | Y | Y | Y | Low |
| *Zager et al [41]* | 1987 | 1 | Y | Y | Y | Y | N | N | Y | Y | Low |

**N, No; UC, Unclear; Y, Yes**
